# IAPP-induced beta cell stress recapitulates the islet transcriptome in type 2 diabetes

**DOI:** 10.1007/s00125-021-05569-2

**Published:** 2021-09-23

**Authors:** Montgomery Blencowe, Allison Furterer, Qing Wang, Fuying Gao, Madeline Rosenberger, Lina Pei, Hiroshi Nomoto, Alex M. Mawla, Mark O. Huising, Giovanni Coppola, Xia Yang, Peter C. Butler, Tatyana Gurlo

**Affiliations:** 1 Department of Integrative Biology and Physiology, University of California, Los Angeles, Los Angeles, CA USA; 2 Molecular, Cellular, and Integrative Physiology Interdepartmental Program, University of California, Los Angeles, Los Angeles, CA USA; 3 Semel Institute for Neuroscience and Human Behavior, University of California, Los Angeles, David Geffen School of Medicine, Los Angeles, CA USA; 4 Larry L. Hillblom Islet Research Center, University of California, Los Angeles, David Geffen School of Medicine, Los Angeles, CA USA; 5 Department of Neurobiology, Physiology and Behavior, College of Biological Sciences, University of California, Davis, Davis, CA USA; 6 Department of Neurology, University of California, Los Angeles, David Geffen School of Medicine, Los Angeles, CA USA

**Keywords:** Beta cell, Cell cycle, Dedifferentiation, Inflammation, Islet amyloid polypeptide, Prediabetes, Protein misfolding, Type 2 diabetes, Unfolded protein response

## Abstract

**Aims/hypothesis:**

Type 2 diabetes is characterised by islet amyloid and toxic oligomers of islet amyloid polypeptide (IAPP). We posed the questions, (1) does IAPP toxicity induce an islet response comparable to that in humans with type 2 diabetes, and if so, (2) what are the key transcriptional drivers of this response?

**Methods:**

The islet transcriptome was evaluated in five groups of mice: beta cell specific transgenic for (1) human IAPP, (2) rodent IAPP, (3) human calpastatin, (4) human calpastatin and human IAPP, and (5) wild-type mice. RNA sequencing data was analysed by differential expression analysis and gene co-expression network analysis to establish the islet response to adaptation to an increased beta cell workload of soluble rodent IAPP, the islet response to increased expression of oligomeric human IAPP, and the extent to which the latter was rescued by suppression of calpain hyperactivation by calpastatin. Rank-rank hypergeometric overlap analysis was used to compare the transcriptome of islets from human or rodent IAPP transgenic mice vs humans with prediabetes or type 2 diabetes.

**Results:**

The islet transcriptomes in humans with prediabetes and type 2 diabetes are remarkably similar. Beta cell overexpression of soluble rodent or oligomer-prone human IAPP induced changes in islet transcriptome present in prediabetes and type 2 diabetes, including decreased expression of genes that confer beta cell identity. Increased expression of human IAPP, but not rodent IAPP, induced islet inflammation present in prediabetes and type 2 diabetes in humans. Key mediators of the injury responses in islets transgenic for human IAPP or those from individuals with type 2 diabetes include STAT3, NF-κB, ESR1 and CTNNB1 by transcription factor analysis and COL3A1, NID1 and ZNF800 by gene regulatory network analysis.

**Conclusions/interpretation:**

Beta cell injury mediated by IAPP is a plausible mechanism to contribute to islet inflammation and dedifferentiation in type 2 diabetes. Inhibition of IAPP toxicity is a potential therapeutic target in type 2 diabetes.

**Graphical abstract:**

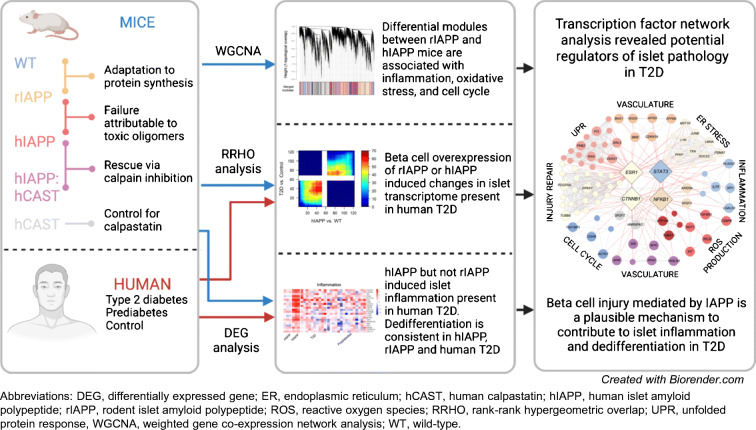

**Supplementary Information:**

The online version contains peer-reviewed but unedited supplementary material available at 10.1007/s00125-021-05569-2.

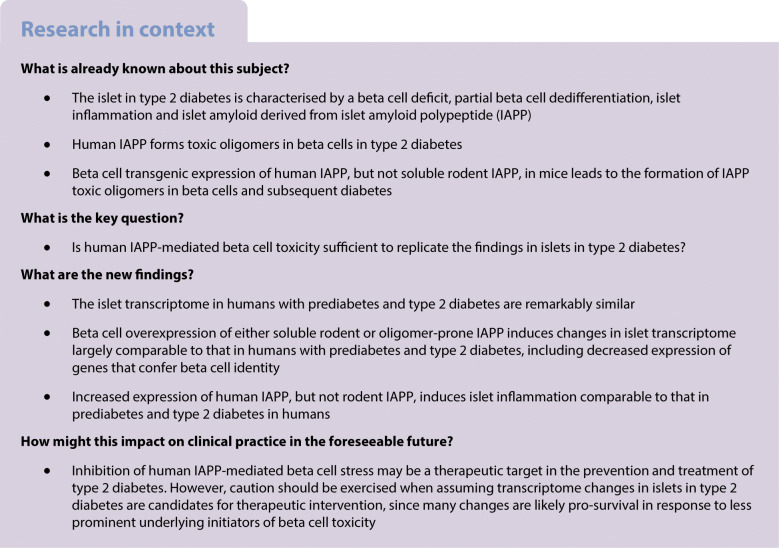



## Introduction

The islet in type 2 diabetes is characterised by islet amyloid derived from islet amyloid polypeptide (IAPP), a protein co-expressed with insulin by beta cells that when misfolded and in aggregate form may contribute to beta cell failure [1–4]. Human IAPP (hIAPP) toxicity is most potently mediated by small intracellular membrane permeant oligomers [5]. Species with amyloidogenic IAPP, such as humans, non-human primates and cats, share vulnerability to type 2 diabetes, while those with non-amyloidogenic IAPP, such as mice and rats, do not [6]. While numerous hypotheses have been put forward to explain the wide-ranging changes in islets in type 2 diabetes [7], there is a consensus that misfolded protein stress induced by toxic oligomers of amyloidogenic proteins initiate these changes in neurodegenerative diseases.

Given the known proximal role of misfolded protein stress in neurodegenerative diseases, and the connection of the risk factors for type 2 diabetes to misfolded protein stress, we hypothesised that hIAPP misfolded protein stress may be a proximal cause of the wide-ranging changes in islets in individuals with type 2 diabetes. Risk factors for type 2 diabetes include insulin resistance [8] and low birthweight [9]. Low birthweight may lead to low adult beta cell mass [10], which together with insulin resistance, predicts a high insulin and IAPP expression rate per beta cell [11]. Beta cell misfolded protein stress is induced when expression of hIAPP per cell exceeds the cellular threshold for clearing misfolded proteins [12]. This threshold declines with ageing [13], a risk factor for both type 2 diabetes and neurodegenerative diseases. hIAPP overexpression in isolated mouse islets in vitro can modify islet gene expression with relevance to type 2 diabetes [14].

In the present study we evaluated the islet transcriptome from a mouse model of beta cell hIAPP toxicity [12] before diabetes onset in order to avoid the confounding effects of hyperglycaemia. To control for the increased burden of IAPP expression, we evaluated the transcriptome from mice overexpressing rodent IAPP (rIAPP) [15]. We then compared the changes in the transcriptome of hIAPP or rIAPP islets to those in humans with prediabetes (defined as an HbA_1c_ level of 42 to 47 mmol/mol [6% to 6.5%]) or type 2 diabetes to establish if the changes in the islet in type 2 diabetes are potentially attributable in part to hIAPP protein misfolding stress.

## Methods

### Mouse models

**Fig. 1:**
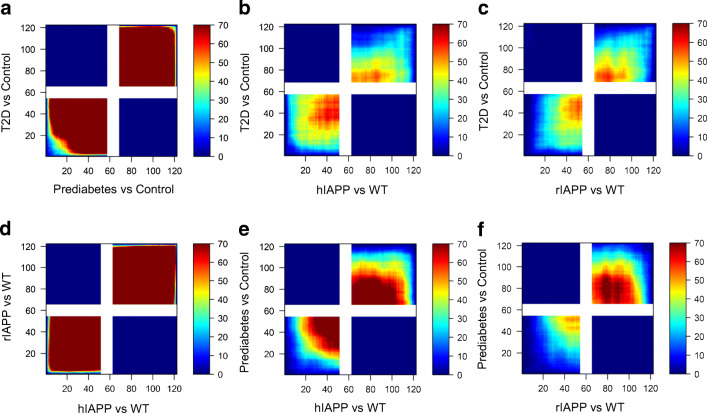
Concordant islet transcriptome changes induced by IAPP in mice and in humans assessed by RRHO analysis. Pixels represent the −log_10_(*p* value) of a hypergeometric test (step size = 110) and are colour-coded to visualise strength and pattern of overlap. The maximally overlapping sets of upregulated genes (signal in lower left quadrant) and downregulated genes (signal in upper right quadrant) are shown. The expression profile of pancreatic islets from prediabetic and T2D donors (relative to normoglycaemic control) are strikingly similar (**a**), as are the expression profiles of pancreatic islets from the IAPP transgenic mouse models (relative to WT, **d**). The expression profile of pancreatic islets from IAPP transgenic mice (relative to WT) is highly concordant with the islet from humans with T2D (**b**, **c**) and prediabetes (**e**, **f**). The proportion of genes subjected to RRHO analysis and concordantly changed are listed in ESM Fig. 2. T2D, type 2 diabetes

Animal studies were approved by the University of California, Los Angeles (UCLA) Office of Animal Research Oversight. The transgenic mice homozygous for human *IAPP* (hIAPP) [12] were originally from Pfizer (available from Jackson Laboratory, Bar Harbor, ME, USA: IMSR cat. no. JAX:008232, RRID:IMSR_JAX:008232) and wild-type FVB (WT) mice (IMSR cat. No. CRL:207, RRID:IMSR_CRL:207) from Charles Rivers Laboratory (Wilmington, MA, USA). The generation of the transgenic mice expressing rodent *Iapp* (rIAPP), human calpastatin (hCAST), and both human *IAPP* and *CAST* (hIAPP:hCAST) on FVB background has been described elsewhere [15, 16]. Mice were bred and maintained at UCLA on 12 h day/night rhythm, Harlan Teklad Rodent Diet 8604 (Placentia, CA, USA) and water ad libitum; diabetes was monitored as described [16]. hIAPP transgenic mice develop diabetes (fasting blood glucose >6.9 mmol/l) after 9 weeks of age, while rIAPP mice remained non-diabetic until 18 weeks of age, the end of observation. See electronic supplementary material (ESM) Fig. 1. Only non-diabetic 9–10-weeks-old male mice were used (ESM Tables 1–4). Expression of IAPP (sum of endogenous and transgenic) is comparable in the rIAPP and hIAPP mice [15]. Mice were either subjected to metabolic studies with fasting blood glucose measurements and GTT, or islets and pancreases were collected for analysis of RNA by bulk islet RNA sequencing (RNA-seq) or qPCR, or analysis of protein levels by western blotting (whole cell lysate in RIPA buffer) or immunostaining (4 μm thick sections of frozen in OCT 4% paraformaldehyde fixed tissue) [16, 17]. See ESM Methods.

### RNA-seq of mouse islets

RNA samples from three mice per group were used for RNA-seq (ESM Table 1). Total RNA was extracted from islet samples (176 ± 11 islets per mouse) using the RNeasy Mini Kit (Qiagen, Germantown, MD, USA). RNA integrity was confirmed using the Agilent Bioanalyzer 2100 (RNA integrity number [RIN] range: 6.8–8.9). RNA-seq libraries were prepared using the TruSeq with Ribo-Zero treatment (Illumina, San Diego, CA, USA) to deplete ribosomal RNA. cDNA libraries were generated using the NuGEN Ovation kit (NuGEN, Redwood City, CA, USA). Illumina’s NextSeq 500 platform was used to generate 75 bp, paired-end reads (64 ± 1.4 million reads per sample). Short reads were aligned to the mouse reference genome build GRCm38 (mm10) using the Spliced Transcripts Alignment to a Reference software (STAR aligner) [18]. Between 65% and 75% (mean 70%) of the reads mapped uniquely to the mouse genome. The HT-Seq package [19] was used to count the number of fragments aligned to known exonic regions. Gene expression was measured as total fragment counts per gene. Sample clustering of islet RNA-seq using multidimensional scaling largely reflected genotype (ESM Fig. 2).

### RNA-seq of human islets

RNA-seq data from human pancreatic islets were downloaded from the Gene Expression Omnibus (GEO) (GSE50244) [20]. Data from 77 samples with available HbA_1c_ values were analysed. Read counts were normalised via the trimmed mean method prior to differential expression analysis using the edgeR package. One type 2 diabetes sample was excluded as an outlier (GSM1216834); therefore 76 samples were included in this manuscript: 51 from normoglycaemic donors with HbA1c levels below 42 mmol/mol (<6%) (HbA_1c_ 5.4 ± 0.1%; BMI 26 ± 0.3; age 56 ± 2; 18 female/33 male), 15 prediabetic donors with HbA_1c_ levels 42 to 47 mmol/mol (6% to 6.5%) (HbA_1c_ 6.1 ± 0.03%; BMI 26 ± 1; age 61 ± 2; 6 female/9 male), and ten donors with type 2 diabetes with HbA_1c_ levels 48 mmol/mol (>6.5%) or higher (HbA_1c_ 7.5 ± 0.3%; BMI 30 ± 1; age 61 ± 3; 6 female/4 male). No additional information about the donors or islet morphology was available.

### RNA-seq data analysis

The RNA-seq data from mouse and human islets were subjected to differential expression analysis [21] to find differentially expressed genes (DEGs) between mouse groups and between human groups. Rank-rank hypergeometric overlap (RRHO) [22] analysis was then used to compare human and mouse gene expression signatures to evaluate between-species similarity and differences (ESM Methods). In order to understand the biological processes informed by the DEGs, we performed functional enrichment analysis (ESM Methods) to identify over-representation of Gene Ontology (GO) terms and Kyoto Encyclopedia of Genes and Genomes (KEGG) pathways [23]. DEGs from mouse islets were further assessed for enrichment of genes associated with Mendelian or common form of diabetes and metabolic syndrome (ESM Methods).

To identify genes with coordinated expression in the form of co-expression modules that are related to diabetes development in mouse models, we utilised weighted gene co-expression network analysis (WGCNA; ESM Methods) [24, 25].

To identify cell types contributing to the gene expression changes, we subjected DEGs and co-expression modules (restricting analysis to genes with module membership >0.5 and false discovery rate [FDR] <0.05) to two types of analysis. First, we carried out cell type marker enrichment analysis (ESM Methods) using PanglaoDB cell marker compendium [26] and GeneOverlap R tool [27]. Second, we applied CibersortX [28] to deconvolute mouse bulk islet RNA-seq data into cell type proportions using single cell RNA-seq mouse islet data from GEO (GSM2230762) as reference (ESM Methods).

To identify shared regulatory factors between islets from hIAPP transgenic mice and islets from humans with type 2 diabetes, we performed transcription factor (TF) network analysis using the Enrichr tool [29] with the TF-Gene Co-occurrence extension. To identify additional non-TF regulators, we utilised the key driver analysis function from the Mergeomics R package [30] to identify regulatory genes for the DEG sets and for the co-expression modules using gene regulatory Bayesian network based on a *χ*^2^ like statistic (ESM Methods).

### Statistical analysis

Statistical analysis was performed as described in the figure legends and ESM.

## Results

### Similarity in islet transcriptome in prediabetes or type 2 diabetes and IAPP overexpressing mice

There is striking concordance of the islet transcriptome between individuals with prediabetes and those with type 2 diabetes (Fig. 1a). Since ~80% of individuals with prediabetes do not develop diabetes [22], this finding implies that a high proportion of the changes in islets in type 2 diabetes are adaptive rather than causal of diabetes. There was also a close concordance of changes in islet gene expression in type 2 diabetes and mouse islets with increased hIAPP or rIAPP expression (Fig. 1b–d, ESM Fig. 2).

Since by design neither the hIAPP or rIAPP mice had diabetes when islets were sampled, we further compared the changes in islet transcriptome in the mouse islets with those in human islets with prediabetes. There was again close concordance in transcriptome in both rIAPP and hIAPP islets with those from individuals with prediabetes (Fig. 1e,f). These findings imply that a more detailed analysis of the transcriptome in response to increased expression of rIAPP and hIAPP might shed light on adaptive vs disease causal changes in type 2 diabetes.

### Islet transcriptome in response to increased beta cell rIAPP or hIAPP expression

**Fig. 2:**
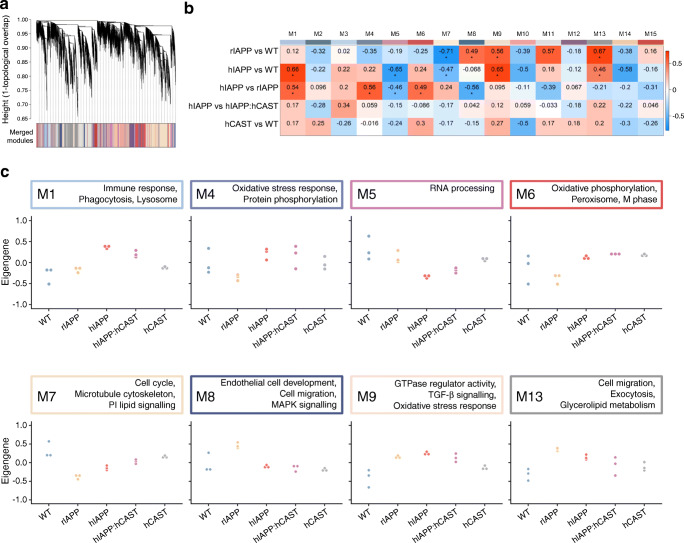
Co-expression network construction and analysis. (**a**) Hierarchical cluster dendrogram generated using all samples grouped genes into 15 distinct co-expression modules (M1–M15, labelled with colours). (**b**) Module-trait relationships were assessed by fitting a generalised linear model based on IAPP and CAST status, then comparing module eigengene (ME)—equivalent to the first principal component of a module—between genotype pairs. Module-level differential expression was tested by one-way, nonparametric ANOVA followed by post hoc Tukey test. Differences in ME expression are presented as a heat map, with significant perturbations denoted (*, *q* < 0.05). (**c**) Trajectory plots of perturbed modules display normalised expression across all samples

**Fig. 3:**
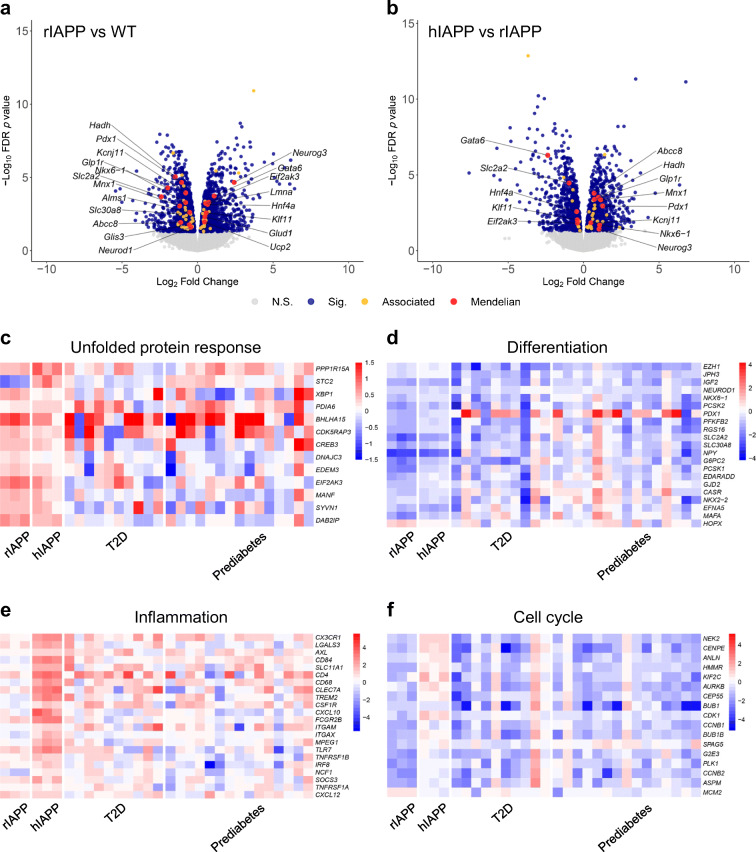
Transcriptomic profiles of adaptation to increased secretory workload, and failure in context of protein misfolding toxicity. Volcano plots show relative expression (Log_2_ fold change) of 15,731 transcripts plotted against the adjusted *p* value from differential expression. (**a**) Comparing rIAPP islets with those of WT defines the expression profile of islets successfully compensating for increased soluble IAPP. Several genes implicated in Mendelian disease are dysregulated (red, labelled), as are genes linked to type 2 diabetes by GWAS (yellow). (**b**) hIAPP islets compared with rIAPP highlights expression dysregulation corresponding to IAPP-derived oligomer toxicity, now controlling for increased beta cell workload. (**c**–**f**) Successful and failed adaptation to increased beta cell secretory pathway burden involves activation of the adaptive UPR, inflammation, altered expression of cell cycle-associated genes, and beta cell dedifferentiation. (**c**) Islets of rIAPP mice show enhanced upregulation of key UPR genes compared with hIAPP. Some UPR-related genes appear to be upregulated in both T2D and prediabetes. (**d**) Increased beta cell workload leads to downregulation of key beta cell function and maturity markers, with rIAPP islets demonstrating more profound ‘dedifferentiation’ than hIAPP islets. (**e**) Increased hIAPP results in transcriptional upregulation of inflammation-associated genes, including several macrophage markers. (**f**) Increased secretory burden drives downregulation of cell cycle-associated genes in islets from individuals with T2D (HbA_1c_ level above 48 mmol/mol [>6.5%]) and donors with prediabetes (HbA_1c_ levels 42 to 47 mmol/mol [6% to 6.5%]), as well as rIAPP islets, but not in hIAPP islets. Data are expressed as a ratio of individual to the mean of WT islets for mouse models, and Control (HbA_1c_ level below 42 mmol/mol [<6%]) for human islets. T2D, type 2 diabetes

To investigate beta cell adaptation to an increased workload of non-amyloidogenic rIAPP expression, we compared the transcriptome of islets from rIAPP vs WT mice. Gene expression was increased in WGCNA modules M8 (Mitogen activated protein kinase [MAPK] signalling), M9 (TGF-β signalling), M13 (cell migration) and decreased in M7 (cell cycle) in rIAPP islets (Fig. 2, Table 1, ESM Fig. 3). Differential expression analysis identified 2731 DEGs (1306 up- and 1425 downregulated in rIAPP islets; FDR < 0.05) (Fig. 3a). Of the 2731 DEGs, 14 are implicated in monogenetic diabetes and 39 are located in genomic regions associated with type 2 diabetes by genome-wide association studies (GWAS), consistent with the overlap between rIAPP and type 2 diabetes by RRHO analysis (Fig. 1c, ESM Fig. 4). Prominently upregulated genes in rIAPP islets include those required for protein synthesis and those that adapt cells to an increased burden of protein folding and quality control, collectively referred to as the unfolded protein response (UPR) (Fig. 3c, ESM Fig. 5, 6).

**Table 1:** Functional characterisation of co-expression modules. Select hub genes with high intramodular connectivity and major biological processes associated with each module by gene set enrichment analysis are reported

Module	Hub genes	Associated biological processes
M1	*Cx3cr1*, *Trf*, *Pla2g7*, *Apoe*, *Trem2*, *Axl*	Immune response, phagocytosis, lysosome
M2	*Tmsb15b2*, *Tk2*, *Micu1*, *Pet112*	Oxidoreductase activity, mitochondria
M3	*Cmklr1*, *Sulf 1*, *Mylk*, *Fap*, *Ndrg2*	ERK1/2 cascade, response to growth factor, regulation of angiogenesis
M4	*Arhgap6*, *Tes*, *Myo1b*, *Por*, *Rcan*	Oxidative stress response, protein phosphorylation
M5	*Utp6*, *Slu7*, *Meis2*, *Arhgef 9*, *Usp11*	RNA processing
M6	*Nell1*, *Atp8a1*, *Capn9*, *Agt*, *Sorl1*	Oxidative phosphorylation, peroxisome, M phase
M7	*Ttbk2*, *Wdr11*, *Ap1s2*, *Maob*, *Dusp10*, *Cdkn2b*	Cell cycle, microtubule cytoskeleton, phosphatidylinositol signalling
M8	*Ptprz1*, *Jam2*, *Mapk4*, *Calb1*, *Bmp3*, *Bcl2*	Endothelial cell development, cell migration and morphogenesis, MAPK signalling
M9	*Carhsp1*, *Lmna*, *Sqstm1*, *Pink1*, *Psma7*, *Lrp10*	GTPase regulator activity, TGF-β signalling, oxidative stress response
M10	*Setd1b*, *Ncor2*, *Nav2*, *Soga1*, *Vamp2*	Transcription regulation, chromatin organisation, insulin secretion
M11	*Svop*, *Pla2g2f*, *Aldoa*, *Usp7*, *Vcp*, *Sec13*	Protein processing in ER, proteasome, cell cycle regulation
M12	*Ints2*, *Zzef 1*, *Gpd2*, *Crhr1*, *Ntrk2*	Protein ubiquitination, chaperonin-mediated protein folding
M13	*Col12a1*, *Mmp2*, *Pld3, Cpt1a*, *Calu*, *Nphs1*	Regulation of cell migration, exocytosis, glycerolipid metabolism
M14	*Zfp758*, *Spopl*, *Nemf*, *Clock*, *Slc1a1*	RNA processing, gene expression, G1/S phase
M15	*Gpatch1*, *Nop58*, *Bub3*, *Pdap1*, *Glis1*, *Bag5*	RNA metabolism and splicing, regulation of cell differentiation

Since there is a considerable overlap in the changes in transcriptome in hIAPP and rIAPP mice (Fig. 1d), and hIAPP but not rIAPP mice develop diabetes, we next compared these transcriptomes to discern changes related to oligomer toxicity vs adaptation to an increased burden of IAPP expression. Five co-expression modules were differentially expressed between hIAPP and rIAPP islets: M1 (inflammation), M4 (oxidative stress) and M6 (cell cycle; cell signalling) expression was increased, while M5 (RNA processing) and M8 (MAPK signalling; cell adhesion) were downregulated in hIAPP (Fig. 2b,c, Table 1). This pattern of changes is consistent with islet inflammation reported in type 2 diabetes [31]. We identified 2011 DEGs between hIAPP and rIAPP islets, with 1031 upregulated and 980 downregulated in hIAPP islets (Fig. 3b). Among these, 13 DEGs have been implicated in monogenetic diabetes and 194 are located in genomic regions associated with type 2 diabetes by GWAS (ESM Fig. 4). A number of interesting trends were found in genes involved in critical pathways associated with type 2 diabetes development (Fig. 3, ESM Fig. 4).

#### UPR

Consistent with the comparable increase in IAPP expression [15], islets in hIAPP mice share a comparable increase in the UPR to rIAPP mice (Fig. 3c). The evaluation of changes in expression of the same genes in humans with type 2 diabetes or prediabetes compared with non-diabetics show consistently upregulated *BHLHA15* (*MIST1*), a potent endoplasmic reticulum (ER) stress-inducible transcriptional regulator upregulated by beta cell Ca^2+^ overload [32].

#### Inflammation

The pronounced signal for inflammation in hIAPP, but not rIAPP islets is also present in human prediabetes and type 2 diabetes (Fig. 3). Consistent with a proinflammatory state, several key cell surface antigens (*Cd4*, *Cd68*, *Cd84*), immune sensors (*Cx3cr1*, *Clec7a*), and macrophage genes (*Axl*, *Slc11a1)* were upregulated in hIAPP islets and in type 2 diabetes. The top hub gene in the M1 module that is most highly activated in hIAPP islets is *Cx3cr1* [33]. This prominence of islet inflammation in hIAPP as compared with rIAPP is further shown by the lack of difference between rIAPP and WT in module M1 (immune response) (Fig. 2b,c).

#### Cell cycle

Activation of cell replication is a key component of injury repair programmes. Cell cycle related genes are increased in hIAPP islets but decreased in rIAPP islets (Fig. 3), likely reflecting the injury-mediated signalling in hIAPP islets vs adaptive UPR in rIAPP islets [34]. Prominent amongst upregulated genes in hIAPP islets are those that enhance cell replication directly (*Cdk1, Cep55)* or indirectly (*Hmmr, Anln*). In contrast, these genes are downregulated in islets from prediabetes and type 2 diabetes, which is perhaps consistent with epigenetic silencing of cell cycle genes in beta cells in adult humans compared with 9-week-old mice [35].

#### Beta cell dedifferentiation

**Fig. 4:**
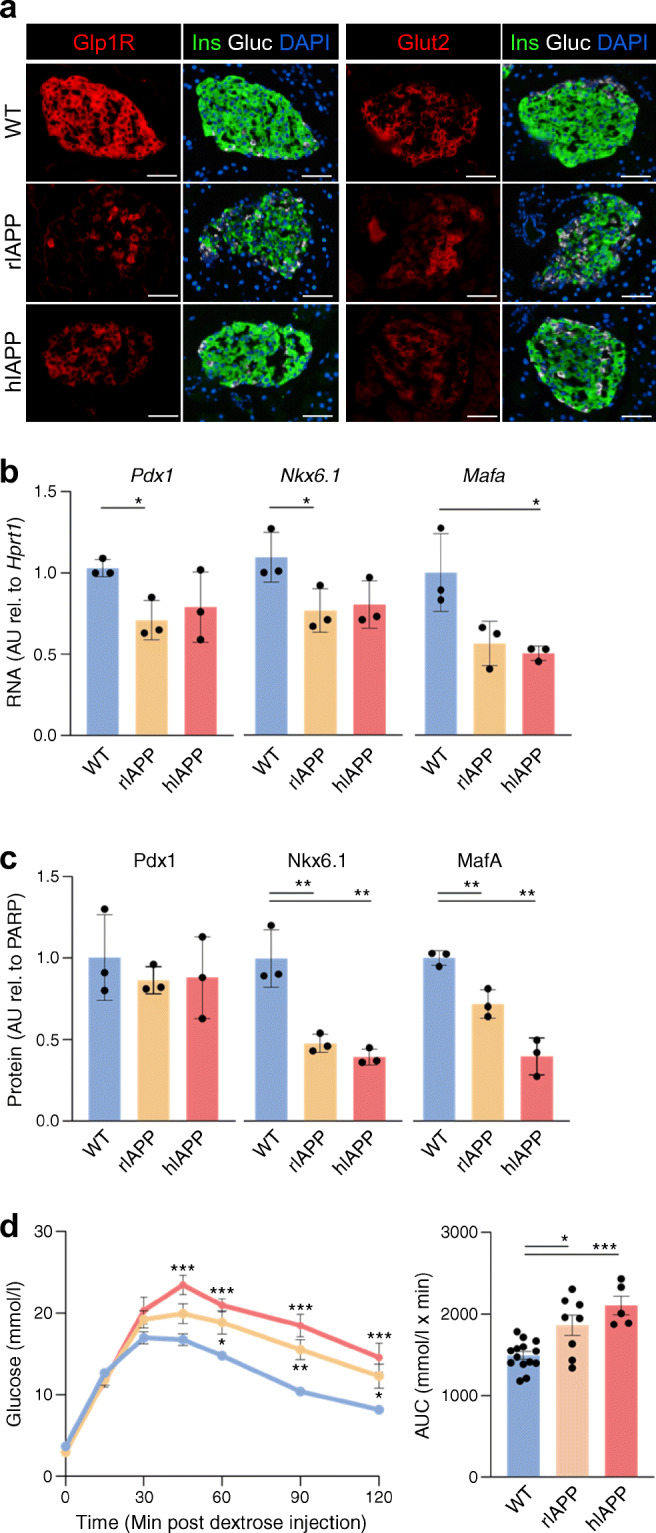
(**a**) Immunohistochemistry staining of islets for Glp1r and Glut2 highlights reduced levels of proteins involved in beta cell secretion. Co-staining for insulin (Ins) and glucagon (Gluc) show comparable cell type composition in rIAPP and hIAPP islets. Scale bar, 50 μm. (**b**, **c**) RNA-seq identified downregulation of the key beta cell TFs (*Nkx6-1*, *Pdx1, Mafa*) in rIAPP and hIAPP islets, and their RNA and protein level expression were tested by qPCR (**b**) and western blotting (**c**), respectively. Data are the mean ± SEM, *n* = 3 in each group, two-tailed Student’s *t* test: *p < 0.05, **p < 0.01. (**d**) IPGTT, 2 mg dextrose/g of body weight after overnight fast; both hIAPP and rIAPP mice display impaired glucose tolerance compared with body weight matched WT, with the greatest effect observed in hIAPP mice. Data are the mean ± SEM, *n* = 5–15 per group; one-way ANOVA followed by post hoc analysis: *p < 0.05, **p < 0.01, ***p < 0.001. Separate islet samples from non-diabetic 9-week-old mice were used to generate the data presented in each panel, and they were different from RNA-seq samples (ESM Tables 1–3)

Genes important for maintaining beta cell differentiation were decreased in islets of both rIAPP and hIAPP mice compared with islets from WT mice (Fig. 3), a pattern reproduced in islets from humans with prediabetes and type 2 diabetes. In mice, beta cell dedifferentiation was confirmed by mRNA and protein analysis (Fig. 4a–c). To establish if this partial dedifferentiation impacts glucose tolerance, we performed IPGTTs. As expected, mice transgenic for hIAPP were glucose intolerant compared with WT mice. Although to a lesser extent, mice transgenic for rIAPP were also glucose intolerant, consistent with the observed partial beta cell dedifferentiation and known action of IAPP to inhibit insulin secretion [36] (Fig. 4d).

### Contribution of calpain hyperactivation to beta cell hIAPP toxicity

Calpain hyperactivation has been widely reported as a mediator of amyloidogenic protein induced cytotoxicity, presumably as a consequence of aberrant Ca^2+^ signalling that results from nonselective ion channel activity of toxic oligomers [37]. Concurrent beta cell-specific overexpression of human calpastatin (*CAST*) (hIAPP:hCAST), which inhibits calpain, delays or prevents diabetes in hIAPP transgenic mice [16].

**Fig. 5:**
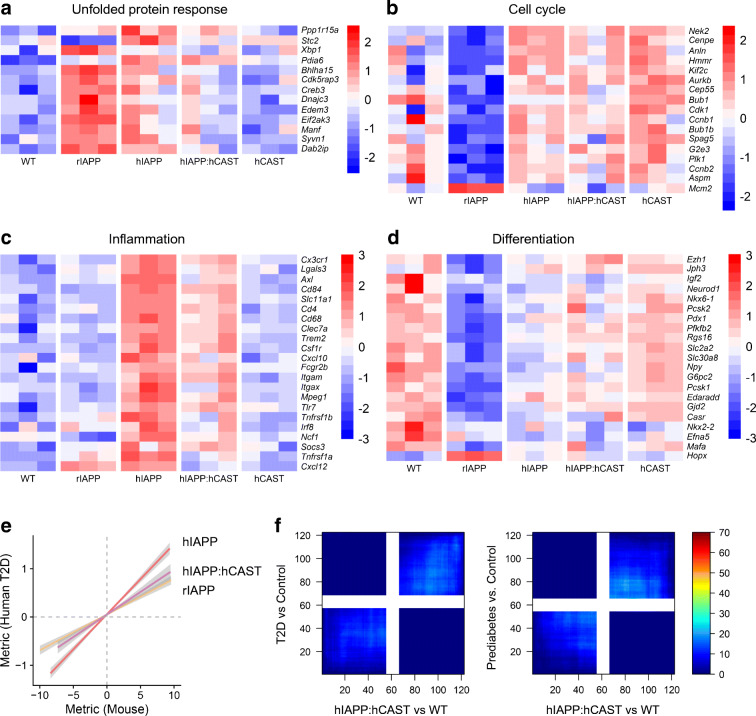
Effect of calpain hyperactivation on gene expression. (**a**–**d**) Increased expression of calpastatin in beta cells from hIAPP mice partially rescues phenotype related to UPR, inflammation, cell cycle and beta cell dedifferentiation. (**e**, **f)** Comparison of islet gene expression profiles affected by IAPP toxicity and calpain hyperactivation with those in human type 2 diabetes. (**e**) Genes measured in two independent experiments are ranked according to degree (nominal *p* value) of differential expression relative to the appropriate control group, multiplied by the sign of the fold change. The type 2 diabetes islet profile is best reflected by the hIAPP islet profile, outperforming the rIAPP and hIAPP:hCAST profiles. (**f**) As an alternative to correlation analysis, we applied the RRHO algorithm to test preservation of IAPP toxicity and calpain hyperactivation signatures in islets from humans with type 2 diabetes and prediabetes. Serial hypergeometric tests were performed at gene rank threshold for two ranked lists. The RRHO map was generated by −log_10_ transformation of the hypergeometric test *p* value (step size = 110), and pixels are colour-coded to visualise strength and pattern of overlap. After accounting for the transcriptomic impact of calpain hyperactivity (hIAPP:hCAST), overlap signal between the islet profiles in type 2 diabetes and prediabetes with the hIAPP mouse model of type 2 diabetes (Fig. 1) significantly decreases, implying a role for calpain in propagating the inflammatory response in pancreatic beta and other cell types in prediabetes and T2D. T2D, type 2 diabetes

Functional enrichment analysis revealed the sets of genes partially rescued by *CAST* overexpression in hIAPP mice were those that mediate UPR and inflammation (Fig. 5). Sustained calpain hyperactivation may activate proinflammatory signalling pathways mediated by NF-κB and endothelial nitric oxide synthase (eNOS) [38].

To further evaluate the role of calpain activation in type 2 diabetes and prediabetes, we correlated the differential expression patterns and found that the type 2 diabetes islet profile is best reflected by the hIAPP islet profile, outperforming the rIAPP and hIAPP:hCAST profiles (Fig. 5e). Similarly, RRHO analysis showed a marked decrease in shared transcriptome between hIAPP and type 2 diabetes islets after introduction of human calpastatin (Fig. 5f vs Fig. 1b,e). These results imply that calpain hyperactivation may play a prominent role in the shared transcriptome between hIAPP and type 2 diabetes.

### Cell type analysis for IAPP misfolded protein stress

**Fig. 6:**
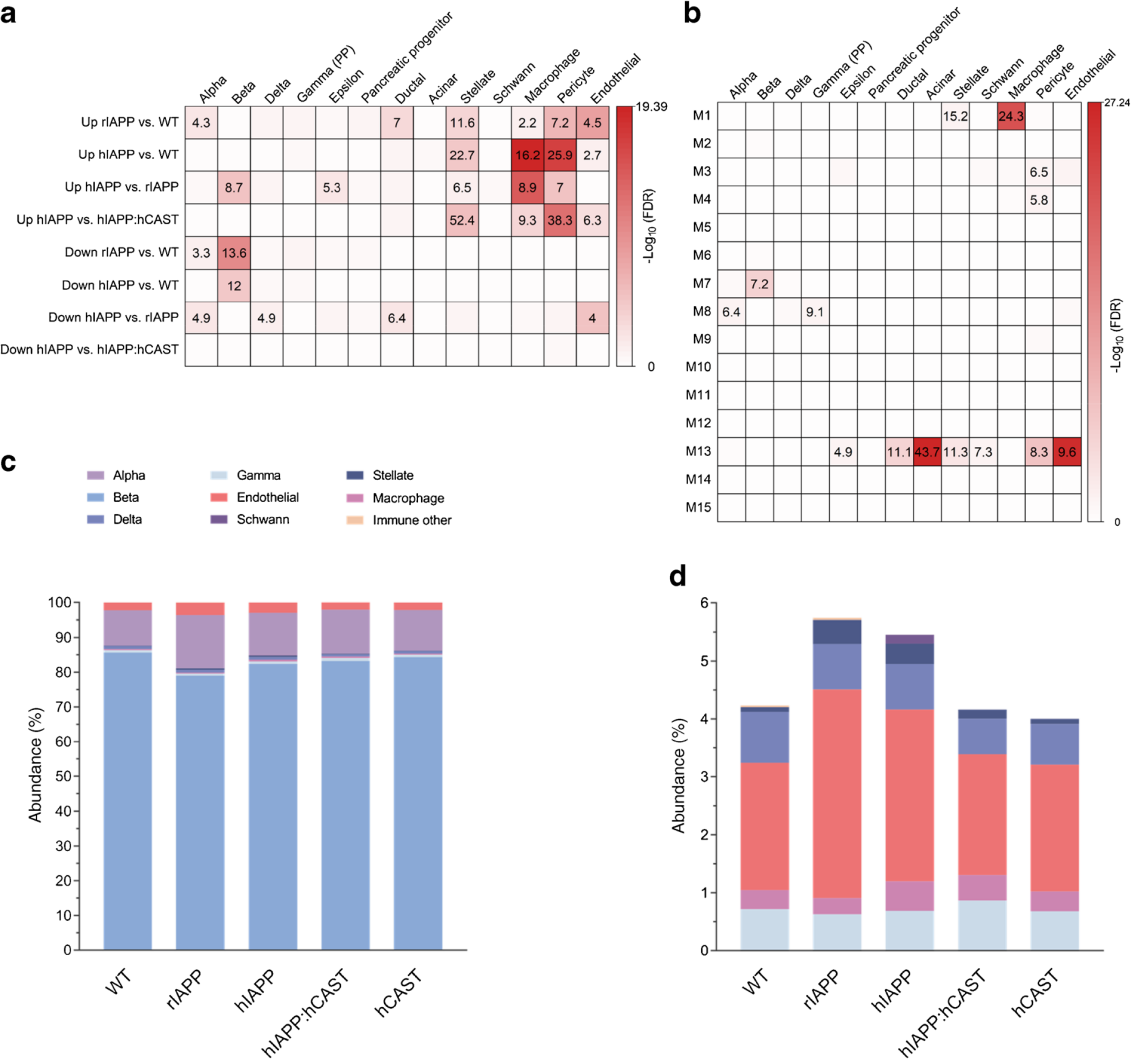
(**a**) Cell type marker enrichment of DEGs highlights substantial contribution of non-endocrine cells toward the composite islet profile in the bulk RNA-seq data. (**b**) Cell type marker enrichment of modules showcases module links with non-endocrine and endocrine cell types. Module enrichment for high-specificity cell type markers was evaluated by Fisher’s exact test. Colour represents the −log_10_ FDR-corrected *p* value, with the OR provided for FDR < 0.05. (**c**) Deconvolution of bulk islet RNA-seq revealed the relative abundance of each cell type captured, highlighting beta cell dominance across the genotypes. (**d**) Magnified view of the deconvolution of bulk islet RNA-seq results on less abundant cell types (alpha and beta cells were excluded) highlighting an increase in endothelial cells, stellate cells and macrophages in hIAPP compared with WT

To explore the cell types that contribute to the transcriptomic signals in the bulk RNA-seq analysis, we conducted cell type marker enrichment analysis of the DEGs and modules as well as cell proportion deconvolution of bulk islet RNA-seq (Fig. 6). These analyses emphasised the impact of beta cell IAPP misfolded protein stress on multiple islet cell types including beta, alpha, endothelial, macrophage and stellate. Both the DEG and module enrichment analysis for cell type markers (Fig. 6a,b) point to a role of stellate cells with marker enrichment for module M13 (cell migration) and downregulated DEGs in hIAPP:hCAST vs hIAPP mice, possibly implicating the role of calpastatin in reducing stellate cell population. The deconvolution results showcase a reduction in beta cell and a rise in alpha cell populations in both rIAPP and hIAPP (Fig. 6c), which alludes to partial beta cell dedifferentiation (Fig. 5d). The increase in endothelial cell populations exemplifies an increase in vascularisation within the islet consistent with response to injury and dedifferentiation. Similarly, the stellate cell populations also follow this trend, and rescue by hIAPP:hCAST shows a reduction in both endothelial and stellate cell (Fig. 6d), which is consistent with reduced inflammation (Fig. 5d). In addition, there is a subtle increase in macrophages in hIAPP (Fig. 6d), matched by macrophage marker enrichment in module M1 (immune response) and in DEGs between hIAPP and rIAPP as well as between hIAPP and hIAPP:hCAST (Fig. 2b,c). These results may explain the differences in inflammation between groups. Complementing this, we found that module M7, associated with beta cells (Fig. 6b), was also enriched for type 2 diabetes GWAS candidate genes (ESM Fig. 4b).

### Prominent regulatory factors shared between hIAPP islets and type 2 diabetes islets

**Fig. 7:**
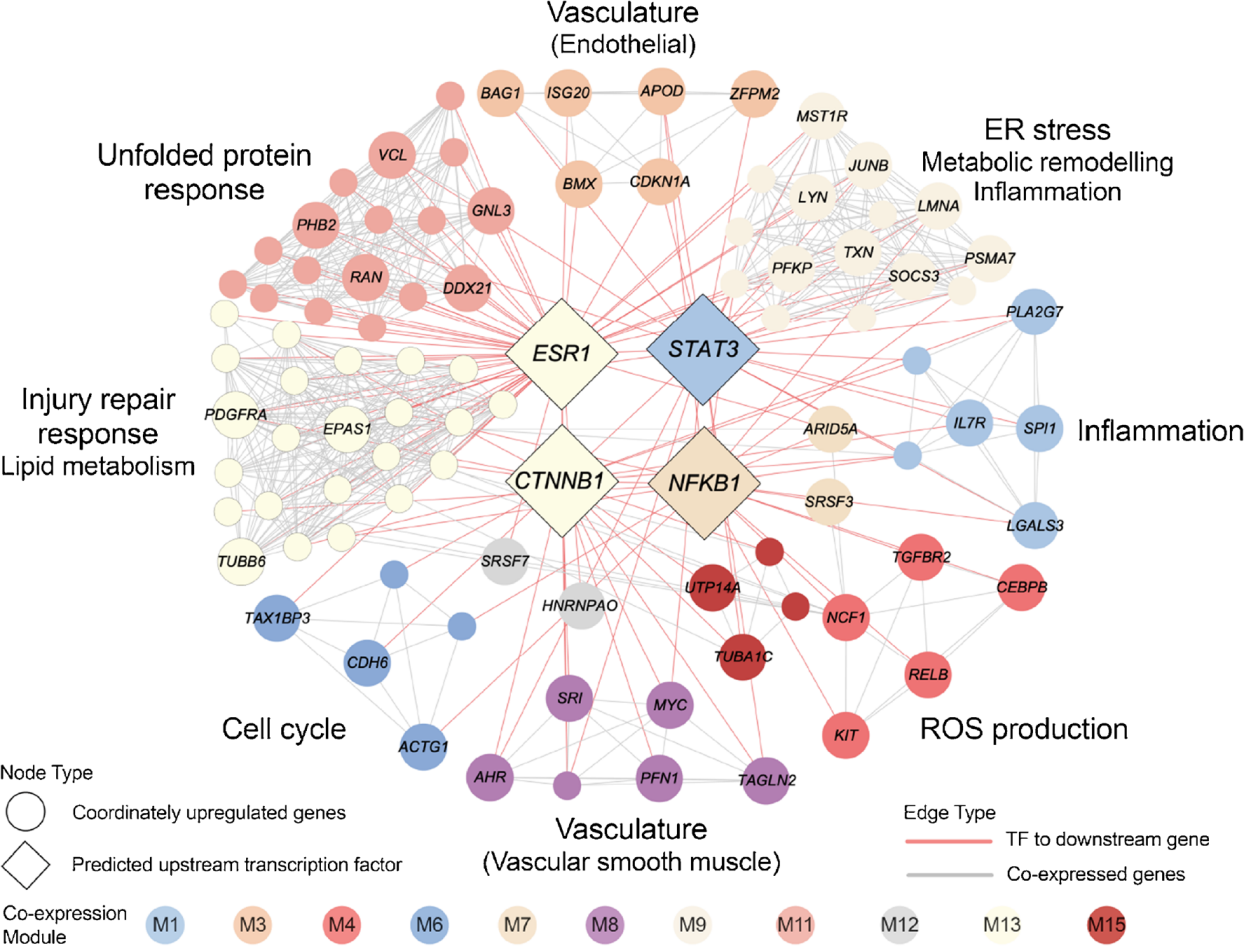
Putative regulatory network of genes co-ordinately upregulated in hIAPP and in human type 2 diabetes islets relative to their respective controls (WT and non-diabetic human islets), identified by RRHO analysis. TF binding sites enrichment analysis identified over-represented upstream TFs including NF-κB1, assigned to beta cell-enriched module (M7), and STAT3, a key regulator of inflammation assigned to macrophage- and stellate cell marker-enriched M1. ESR1 and CTNNB1 are both implicated in beta cell stress/survival signalling. Node colour reflects co-expression module assignment. Edges represent experimentally validated transcription factor-target relationships (red) and intramodular co-expression (light grey)

Having established that hIAPP-induced beta cell toxicity in mouse islets results in an islet transcriptome mimicking that of human islets in type 2 diabetes, we evaluated regulatory cascades in hIAPP and type 2 diabetes-associated transcriptomic alterations by TF network and non-TF gene network analysis. TF network analysis uncovered four upstream hub transcriptional factors *NF-κB*, *ESR1*, *STAT3* and *CTNNB1* active in both type 2 diabetes and hIAPP islets (Fig. 7). NF-κB activation has been implicated as a core transcriptional mediator of neuronal cell inflammatory responses to amyloidogenic misfolded stress in neurons and can be protective or contribute to toxicity of injured beta cells [39] [40] and is activated by aberrant Ca^2+^ signalling and calpain hyperactivation [41]. NF-κB1 was assigned as an upstream regulator to co-expression module M7 that is enriched for beta cell markers (Fig. 6b) and type 2 diabetes GWAS candidate genes (ESM Fig. 4b). NF-κB is a known target of calpain, consistent with calpain hyperactivation in beta cells in hIAPP mouse islets and type 2 diabetes. STAT3 is activated by aberrant Ca^2+^ signalling and has been reported as a key regulator of inflammation in neurodegenerative diseases [42]. STAT3 and NF-κB cooperate as transcriptional regulators to induce angiogenesis, cellular proliferation and pro-survival metabolic remodelling, the latter through activation of hypoxia-inducible factor 1 α (HIF1α) [43]. CTNNB1 encodes β-catenin that has been implicated in tissue repair and regeneration responses in gut and beta cells, inducing cell proliferation, cell migration repair of cytoskeleton and regulation of intracellular Ca^2+^ dynamics [34]. ESR1 signalling is protective of beta cells in response to injury in both human and mouse islets [44].

**Fig. 8:**
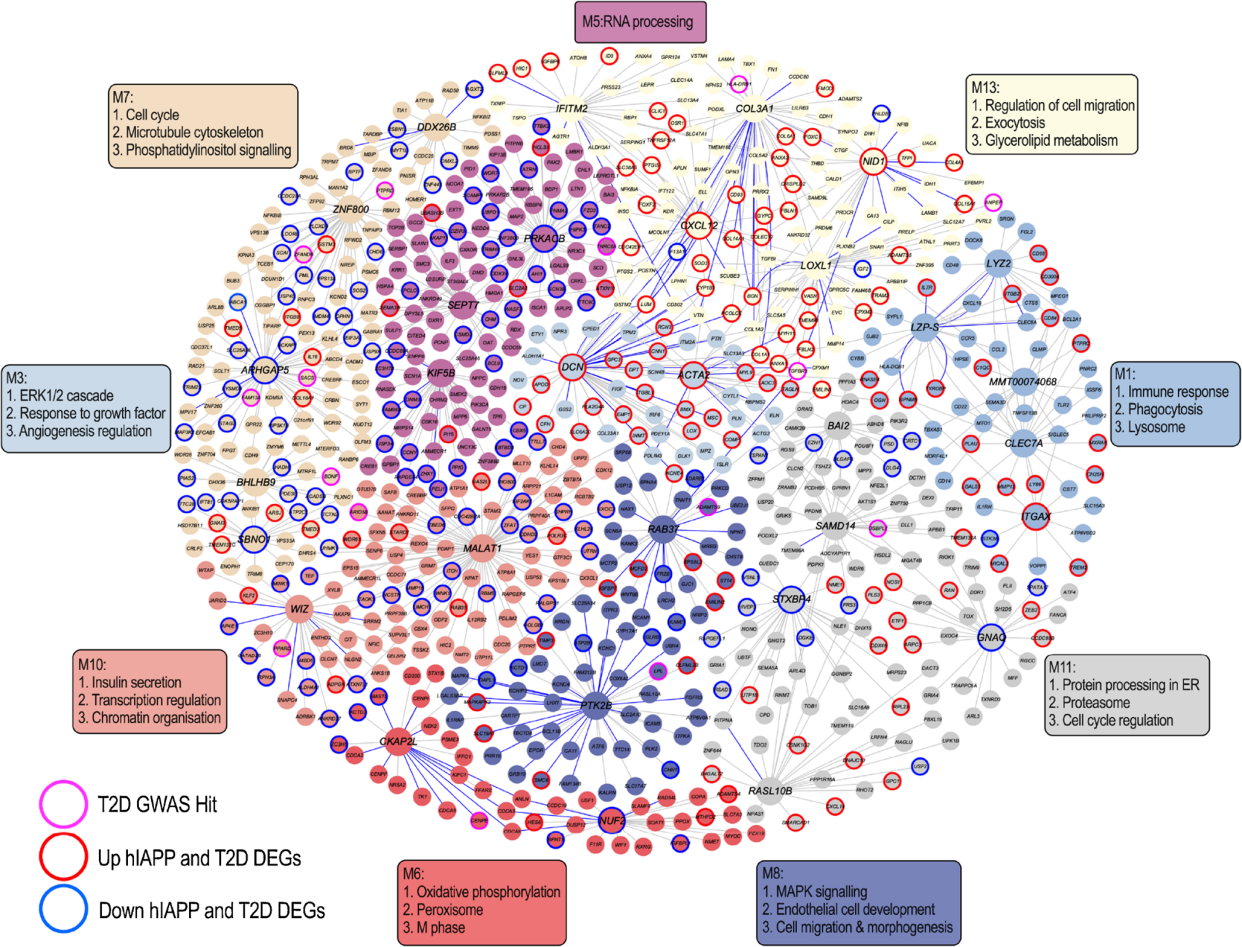
Bayesian gene regulatory network illustrating the co-expression module interconnectivity and highlighting key genes potentially important in driving those processes (indicated by the larger node size). T2D GWAS hits (association *p* < 5 × 10^−8^) are highlighted on the network with pink rings around the nodes, upregulated hIAPP and T2D DEGs are highlighted with red rings, and downregulated hIAPP and T2D DEGs are highlighted by blue rings. T2D, type 2 diabetes

Complementary to the TF network analysis, which is not tissue specific, we utilised an islet gene regulatory network constructed using population-based genetic and transcriptomic datasets, which captures network regulators that are not necessarily TFs. Here, we uncover the interconnectivity between modules/processes for their role in islet pathogenesis and their associated regulatory genes (Fig. 8; ESM Fig. 7). With stellate and endothelial cell markers enriched for M13 (Fig. 6b), hub genes such as *COL3A1* (extracellular matrix), *NID1* (wound healing) and *CXCL12* (leucocyte trafficking, angiogenesis and vascular repair) are plausible regulatory genes underlying stellate and endothelial cell contribution to islet pathogenesis. Moreover, module M7, which is enriched for beta cell markers (Fig. 6b) and type 2 diabetes GWAS candidates (Fig. 8; ESM Fig. 4b), highlights hub genes such as *ZNF800*, which is closely associated with *PAX4* [45], important in the development and differentiation of beta cells.

## Discussion

**Fig. 9:**
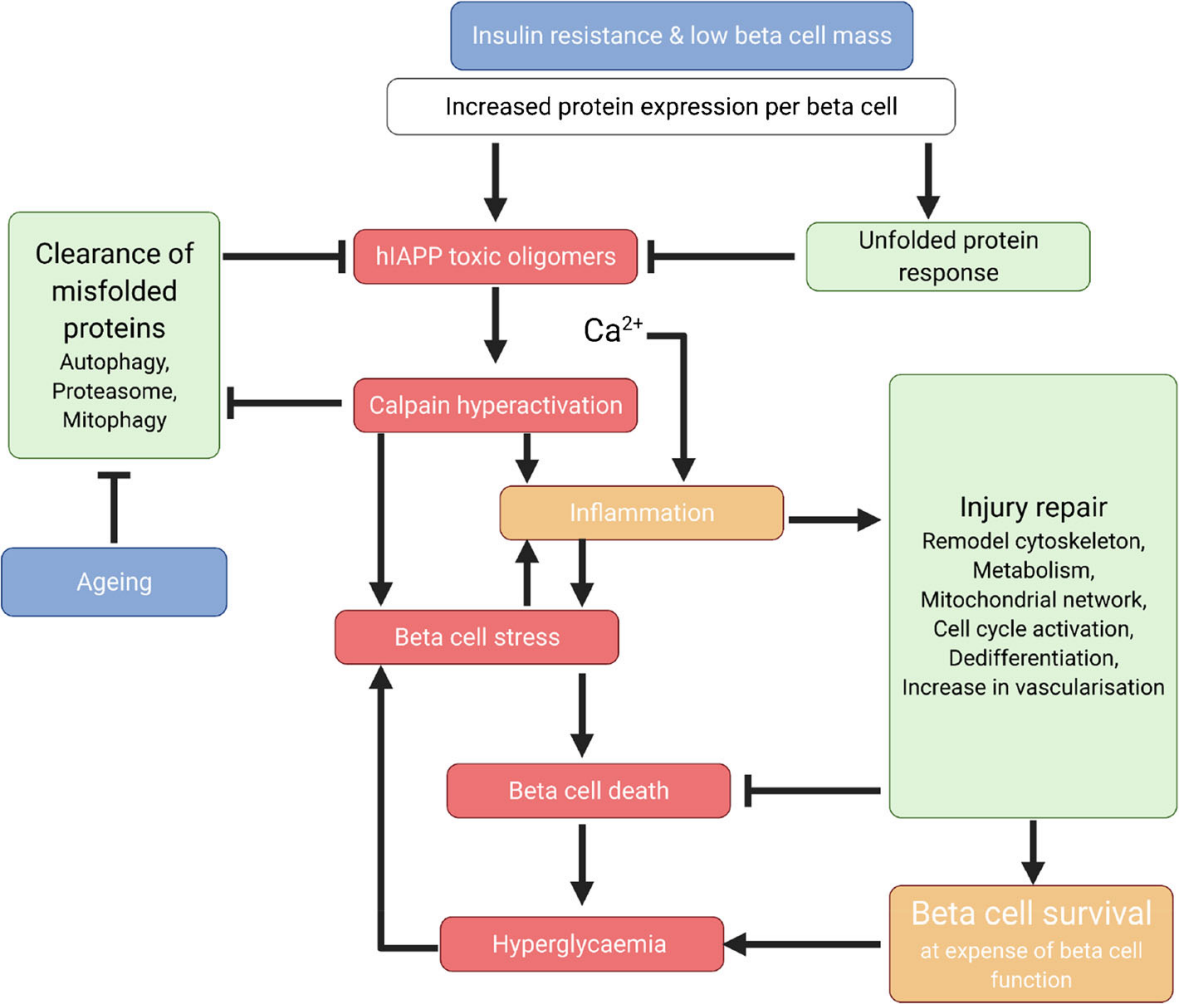
Proposed model of IAPP toxicity in type 2 diabetes in relation to the major risk factors insulin resistance and a low innate beta cell mass, which result in very high expression levels of aggregate toxic oligomer-prone IAPP per beta cell in humans. Clearance of misfolded IAPP by autophagy and proteasome declines with ageing. Increased IAPP and insulin expression induces the protective UPR. Membrane permeant toxic oligomers of IAPP lead to aberrant Ca^2+^ signalling that induces injury inflammatory responses directly and via calpain hyperactivation. These initially activate conserved pro-survival injury repair signalling responses that prolong beta cell survival at the expense of function. However, the adverse actions of calpain hyperactivation on defence against proteotoxicity exacerbates IAPP toxicity, gradually overcoming pro-survival responses

The synthesis, folding and processing of insulin is close to the limit of the biosynthetic capacity of beta cells [46]. hIAPP is highly oligomer prone and readily assembles into membrane permeant toxic oligomers if the rate of expression exceeds the capacity of the cell to fold and traffic newly expressed protein. The propensity of IAPP to form toxic oligomers defines the relative vulnerability of a species to develop type 2 diabetes. Taken together, these observations suggest protein misfolding may contribute to beta cell failure leading to type 2 diabetes under conditions of insulin resistance (Fig. 9).

A striking finding from the studies is the high degree of coordinate transcriptome between islets of humans with prediabetes and type 2 diabetes. Since most individuals with prediabetes do not progress to diabetes, these findings imply much of the islet transcriptome in type 2 diabetes may reflect protective pro-survival changes. Furthermore, we found a close overlap in transcriptome between islets from mice with beta cell overexpression of rIAPP and islets from humans with either prediabetes or type 2 diabetes. Since rIAPP overexpressing mice do not develop diabetes, these findings imply that much of the islet transcriptome in prediabetes and type 2 diabetes reflects adaptive changes to increased beta cell protein synthesis. The importance of successful adaptation of beta cells to increased expression of secretory protein is further illustrated by the large number of genes linked to vulnerability to type 2 diabetes, by GWAS or Mendelian association, that were differentially expressed in rIAPP compared with WT islets.

To better understand the potential role of hIAPP toxicity in beta cell failure and loss in type 2 diabetes, we evaluated the transcriptome of islets in mice overexpressing hIAPP with that in islets of humans with type 2 diabetes. In both hIAPP islets and islets from individuals with type 2 diabetes, there was a strong inflammatory signal, consistent with an ongoing injury response. Network analysis reveals that shared key pro-survival gene networks (Fig. 7) are activated in hIAPP islets and islets from individuals with type 2 diabetes, consistent with the slow progression of beta cell loss in type 2 diabetes. Notably, a decrease in expression of genes that confer beta cells their identity (dedifferentiation) is apparent in both rIAPP mouse and prediabetes human islets, implying that beta cell dedifferentiation maybe a pro-survival adaptation.

By comparing the successful adaptation of beta cells to rIAPP vs hIAPP overexpression, the most prominent difference was the increased expression of genes ascribed to inflammation that were markedly increased in hIAPP islets and type 2 diabetes but only modestly increased in rIAPP islets and in prediabetes. Type 2 diabetes is a heterogeneous disease and there are likely multiple pathways to beta cell toxicity, including toxic actions of lipids and hyperglycaemia [47]. In a recent partial pancreatectomy study in rats, the resulting marked decrease in beta cell mass and modest hyperglycaemia induced many of the same signals observed in response to beta cell hIAPP toxicity in the present study, including inflammation and partial beta cell differentiation [48]. In an in vitro acute injury study of human islets exposed to glucolipotoxicity, there was also some overlap with islets from humans with type 2 diabetes by RRHO analysis [49], but the overlap was weaker compared with IAPP overexpression in the current study. The difference could be due to in vivo vs in vitro conditions or intrinsic differences between IAPP and glucolipotoxicity.

A limitation of the current study is the use of whole pancreatic islets for RNA-seq, which masks the cell types contributing to the transcriptional signals. Although we used cellular deconvolution and cell marker enrichment methods to mitigate this issue, single cell RNA-seq will be of added value in future studies to confirm the predicted cell type contributions from our deconvolution analysis. In addition, follow-up on the various genes found to be contributing to hIAPP beta cell toxicity through knockdown or overexpression studies will be of use to further confirm their causal role in disease*.* Another limitation of the present study is the potential bias caused by use of an inbred mouse FVB strain.

In conclusion, the present studies suggest that much of the islet transcriptome in type 2 diabetes is adaptive to the increased beta cell burden of protein synthesis and folding. Beta cell hIAPP toxicity induces a prominent islet inflammatory response, consistent with that observed in type 2 diabetes, implying protein misfolding stress may serve to initiate or contribute to beta cell injury in type 2 diabetes. There are also shared pro-survival gene networks in hIAPP and type 2 diabetes islets.

Taken together these studies suggest caution should be taken in interpreting transcriptome changes in islets in type 2 diabetes as therapeutic targets since many, if not most, of these changes are likely pro-survival adaptations. Strategies to suppress IAPP expression warrant further investigation due to the mounting evidence to suggest its role in type 2 diabetes pathogenesis.

## Supplementary Information

ESM (PDF 2.56 mb)

## Abbreviations

DEG: Differentially expressed gene
ER: Endoplasmic reticulum
FDR: False discovery rate
GEO: Gene expression omnibus
GWAS: Genome-wide association studies
hCAST: Human calpastatin
hIAPP: Human islet amyloid polypeptide
IAPP: Islet amyloid polypeptide
MAPK: Mitogen activated protein kinase
rIAPP: Rodent islet amyloid polypeptide
RNA-seq: RNA sequencing
RRHO: Rank-rank hypergeometric overlap
TF: Transcription factor
UCLA: University of California, Los Angeles
UPR: Unfolded protein response
WGCNA: Weighted gene co-expression network analysis
WT: Wild-type

## References

[1] Jurgens CA, Toukatly MN, Fligner CL (2011). β-Cell loss and β-cell apoptosis in human type 2 diabetes are related to islet amyloid deposition. Am J Pathol.

[2] Hull RL, Westermark GT, Westermark P, Kahn SE (2004). Islet amyloid: a critical entity in the pathogenesis of type 2 diabetes. J Clin Endocrinol Metab.

[3] Kahn SE, Andrikopoulos S, Verchere CB (1999). Islet amyloid: a long-recognized but underappreciated pathological feature of type 2 diabetes. Diabetes.

[4] Cooper GJ, Willis AC, Clark A, Turner RC, Sim RB, Reid KB (1987). Purification and characterization of a peptide from amyloid-rich pancreases of type 2 diabetic patients. Proc Natl Acad Sci.

[5] Janson J, Ashley RH, Harrison D, McIntyre S, Butler PC (1999). The mechanism of islet amyloid polypeptide toxicity is membrane disruption by intermediate-sized toxic amyloid particles. Diabetes.

[6] O'Brien TD, Butler PC, Westermark P, Johnson KH (1993). Islet amyloid polypeptide: a review of its biology and potential roles in the pathogenesis of diabetes mellitus. Vet Pathol.

[7] Halban PA, Polonsky KS, Bowden DW (2014). beta-cell failure in type 2 diabetes: postulated mechanisms and prospects for prevention and treatment. J Clin Endocrinol Metab.

[8] Haffner SM (1998). Epidemiology of type 2 diabetes: risk factors. Diabetes Care.

[9] Barker DJ, Hales CN, Fall CH, Osmond C, Phipps K, Clark PM (1993). Type 2 (non-insulin-dependent) diabetes mellitus, hypertension and hyperlipidaemia (syndrome X): relation to reduced fetal growth. Diabetologia.

[10] Sasaki H, Saisho Y, Inaishi J et al (2020) Associations of birthweight and history of childhood obesity with beta cell mass in Japanese adults. Diabetologia. 10.1007/s00125-020-05127-2 10.1007/s00125-020-05127-2 PMC7228916 32239263

[11] Costes S, Langen R, Gurlo T, Matveyenko AV, Butler PC (2013). beta-cell failure in type 2 diabetes: a case of asking too much of too few?. Diabetes.

[12] Janson J, Soeller WC, Roche PC (1996). Spontaneous diabetes mellitus in transgenic mice expressing human islet amyloid polypeptide. Proc Natl Acad Sci U S A.

[13] Vilchez D, Saez I, Dillin A (2014). The role of protein clearance mechanisms in organismal ageing and age-related diseases. Nat Commun.

[14] Hogan MF, Ziemann M, Harikrishnan KN (2019). RNA-seq-based identification of Star upregulation by islet amyloid formation. Protein Eng Des Sel.

[15] Huang CJ, Haataja L, Gurlo T (2007). Induction of endoplasmic reticulum stress-induced beta-cell apoptosis and accumulation of polyubiquitinated proteins by human islet amyloid polypeptide. Am J Physiol Endocrinol Metab.

[16] Gurlo T, Costes S, Hoang JD, Rivera JF, Butler AE, Butler PC (2016). beta cell-specific increased expression of calpastatin prevents diabetes induced by islet amyloid polypeptide toxicity. JCI Insight.

[17] Rivera JF, Costes S, Gurlo T, Glabe CG, Butler PC (2014). Autophagy defends pancreatic beta cells from human islet amyloid polypeptide-induced toxicity. J Clin Invest.

[18] Dobin A, Davis CA, Schlesinger F (2013). STAR: ultrafast universal RNA-seq aligner. Bioinformatics.

[19] Anders S, Pyl PT, Huber W (2015). HTSeq--a Python framework to work with high-throughput sequencing data. Bioinformatics.

[20] Fadista J, Vikman P, Laakso EO (2014). Global genomic and transcriptomic analysis of human pancreatic islets reveals novel genes influencing glucose metabolism. Proc Natl Acad Sci U S A.

[21] Law CW, Chen Y, Shi W, Smyth GK (2014). voom: precision weights unlock linear model analysis tools for RNA-seq read counts. Genome Biol.

[22] Cahill KM, Huo Z, Tseng GC, Logan RW, Seney ML (2018). Improved identification of concordant and discordant gene expression signatures using an updated rank-rank hypergeometric overlap approach. Sci Rep.

[23] Ogata H, Goto S, Fujibuchi W, Kanehisa M (1998). Computation with the KEGG pathway database. Biosystems.

[24] Langfelder P, Horvath S (2007). Eigengene networks for studying the relationships between co-expression modules. BMC Syst Biol.

[25] Langfelder P, Horvath S (2008). WGCNA: an R package for weighted correlation network analysis. BMC Bioinformatics.

[26] Franzen O, Gan LM, Bjorkegren JLM (2019) PanglaoDB: a web server for exploration of mouse and human single-cell RNA sequencing data. Database (Oxford) 2019. 10.1093/database/baz046 10.1093/database/baz046 PMC6450036 30951143

[27] Shen L (2014) GeneOverlap: an R package to test and visualize gene overlaps. R Package

[28] Newman AM, Steen CB, Liu CL (2019). Determining cell type abundance and expression from bulk tissues with digital cytometry. Nat Biotechnol.

[29] Chen EY, Tan CM, Kou Y (2013). Enrichr: interactive and collaborative HTML5 gene list enrichment analysis tool. BMC Bioinformatics.

[30] Shu L, Zhao Y, Kurt Z (2016). Mergeomics: multidimensional data integration to identify pathogenic perturbations to biological systems. BMC Genomics.

[31] Eguchi K, Nagai R (2017). Islet inflammation in type 2 diabetes and physiology. J Clin Invest.

[32] Hess DA, Strelau KM, Karki A (2016). MIST1 links secretion and stress as both target and regulator of the unfolded protein response. Mol Cell Biol.

[33] Riopel M, Seo JB, Bandyopadhyay GK (2018). Chronic fractalkine administration improves glucose tolerance and pancreatic endocrine function. J Clin Invest.

[34] Keefe MD, Wang H, De la OJ, Khan A, Firpo MA, Murtaugh LC (2012). beta-catenin is selectively required for the expansion and regeneration of mature pancreatic acinar cells in mice. Dis Model Mech.

[35] Tschen SI, Dhawan S, Gurlo T, Bhushan A (2009). Age-dependent decline in beta-cell proliferation restricts the capacity of beta-cell regeneration in mice. Diabetes.

[36] Young A (2005). Inhibition of insulin secretion. Adv Pharmacol.

[37] Ferreira A (2012). Calpain dysregulation in Alzheimer’s disease. ISRN Biochem.

[38] Ji J, Su L, Liu Z (2016). Critical role of calpain in inflammation. Biomed Rep.

[39] Eldor R, Yeffet A, Baum K (2006). Conditional and specific NF-kappaB blockade protects pancreatic beta cells from diabetogenic agents. Proc Natl Acad Sci U S A.

[40] Jones SV, Kounatidis I (2017). Nuclear factor-kappa B and Alzheimer disease, unifying genetic and environmental risk factors from cell to humans. Front Immunol.

[41] Lilienbaum A, Israel A (2003). From calcium to NF-kappa B signaling pathways in neurons. Mol Cell Biol.

[42] Reichenbach N, Delekate A, Plescher M et al (2019) Inhibition of Stat3-mediated astrogliosis ameliorates pathology in an Alzheimer’s disease model. EMBO Mol Med 11(2). 10.15252/emmm.201809665 10.15252/emmm.201809665 PMC6365929 30617153

[43] Grivennikov SI, Karin M (2010). Dangerous liaisons: STAT3 and NF-kappaB collaboration and crosstalk in cancer. Cytokine Growth Factor Rev.

[44] Zhou Z, Ribas V, Rajbhandari P (2018). Estrogen receptor alpha protects pancreatic beta-cells from apoptosis by preserving mitochondrial function and suppressing endoplasmic reticulum stress. J Biol Chem.

[45] Kato N (2013). Insights into the genetic basis of type 2 diabetes. J Diabetes Investig.

[46] Rege NK, Liu M, Yang Y (2020). Evolution of insulin at the edge of foldability and its medical implications. Proc Natl Acad Sci U S A.

[47] Prentki M, Matschinsky FM, Madiraju SR (2013). Metabolic signaling in fuel-induced insulin secretion. Cell Metab.

[48] Ebrahimi AG, Hollister-Lock J, Sullivan BA, Tsuchida R, Bonner-Weir S, Weir GC (2020). Beta cell identity changes with mild hyperglycemia: implications for function, growth, and vulnerability. Mol Metab.

[49] Marselli L, Piron A, Suleiman M (2020). Persistent or transient human beta cell dysfunction induced by metabolic stress: specific signatures and shared gene expression with type 2 diabetes. Cell Rep.

