# Supplementary material for: IAPP-induced beta cell stress recapitulates the islet transcriptome in type 2 diabetes
Source: Diabetologia. 2021 Sep 23;65(1):173–87. doi: 10.1007/s00125-021-05569-2 (PMC8660728; doi:10.1007/s00125-021-05569-2)
Supplement: Supplementary file 1 — (PDF 2.56 mb) [file 125_2021_5569_MOESM1_ESM.pdf]

## ELECTRONIC SUPPLEMENTARY MATERIALS

# IAPP induced beta cell stress recapitulates the islet transcriptome in type 2 diabetes

Montgomery Blencowe[1,2], Allison Furterer[3,4], Qing Wang[3], Fuying Gao[3], Madeline Rosenberger[4], Lina Pei[4], Hiroshi Nomoto[4], Alex M Mawla[5], Mark O Huising[5], Giovanni Coppola[3,6], Xia Yang[1,2], Peter C. Butler[4], Tatyana Gurlo[4*]

## ESM Methods

### Mouse models

Animal studies were approved by the UCLA Office of Animal Research Oversight. The transgenic mice homozygous for human *IAPP* (hIAPP) [1] were originally from Pfizer (available from Jackson Laboratory, Bar Harbor, ME, USA: IMSR cat. no. JAX:008232, RRID:IMSR_JAX:008232) and wild-type FVB (WT) mice (IMSR cat. No. CRL:207, RRID:IMSR_CRL:207) from Charles Rivers Laboratory (Wilmington, MA, USA). The generation of the transgenic mice expressing rodent *IAPP* (rIAPP), human calpastatin (hCAST), and both human *IAPP* and *CAST* (hIAPP:hCAST) on FVB background was described elsewhere [2, 3]. Mice were bred and maintained at UCLA on 12-h day/night rhythm, Harlan Teklad Rodent Diet 8604 (Placentia, CA, USA), water ad libitum; diabetes was monitored as described [2]. hIAPP transgenic mice develop diabetes (fasting blood glucose > 6.9mmol/l) after 9 weeks of age, while rIAPP mice remained non-diabetic until 18 weeks of age, the end of observation. See ESM Fig. 1. Only non-diabetic 9-10-weeks-old male mice were used (ESM Tables 1-4). Expression of IAPP (sum of endogenous and transgenic) is comparable in the rIAPP and hIAPP mice [3]. Mice were either subjected to metabolic studies with fasting blood glucose measurements and glucose tolerance test, or islets and pancreases were collected for analysis of RNA by bulk islet RNA sequencing (RNA-seq) or qPCR, or analysis of protein levels by western blot or immunofluorescence [2, 4].

## Metabolic studies

Fasted weight and blood glucose values were obtained in the morning after overnight fasting (clean cages and bedding, no food, water ad libitum).

Blood glucose values were measured in a tail-vein blood sample with a FreeStyle Freedom Lite Glucometer (Abbott, Alameda, CA). The pancreases were then rapidly dissected from euthanized animals for morphological studies, or islets were isolated (see below). A glucose tolerance test was performed after overnight fast. Blood glucose was measured before IP injection of 2 g/kg of dextrose, and 15, 30, 45, 60, 90 and 120 min after.

## Islet isolation

Islets were isolated by collagenase digestion and manually picked [4]; frozen in RLT buffer (Qiagen, Germantown, MD, USA) supplemented with bME (Sigma-Aldrich, St. Louis, MO, USA) and stored at -80°C until RNA isolation for RNA sequencing or gene expression analysis by qPCR.

For protein analysis, islets were lysed in ice-cold RIPA buffer supplemented with protease-phosphatase inhibitor cocktail (Cell Signaling Technologies 5872, Danvers, MA, USA), sonicated, and spun at 10,000 *g* at 4°C for 10min. Supernatant was stored at −80°C until western blot analysis.

## qPCR

RNA isolation was performed using Qiagen RNA Mini kit according to the manufacturer instructions. Single stranded cDNA was prepared using Superscript III First-Strand Synthesis System Kit (Invitrogen, Carlsbad, CA, USA). Real time quantitative PCR was performed using

FAST SYBRGreen master mix and ABI7900HT equipment (Applied Biosystems™, Foster City, CA, USA). The following mouse primers were used:

*Mafa* - CTCCAGAGCCAGGTGGAG and GTACAGGTCCCGCTCCTTG; *Pdx1* - CGGCTGAGCAAGCTAAGGTT and TGGAAGAAGCGCTCTCTTTGA; *Nkx6.1* – CCCGGAGTGATGCAGAGT and GAACGTGGGTCTGGTGTGTT; housekeeping gene *Hprt1* – CTCCTCAGACCGCTTTTTGC and TAACCTGGTTCATCATCGCTAATC.

**Western blotting**

Protein concentration was measured using DC protein assay (Bio-Rad, Irvine, CA, USA). Proteins (20µg per lane) were separated on 4-12% BisTris NuPAGE gels (Invitrogen, Carlsbad, CA, USA) and blotted onto a PVDF membrane (Pall, Ann Arbor, MI, USA). Membranes were blocked in 5% milk (BioRad, Hercules, CA, USA), incubated overnight with primary antibodies diluted in the antibody buffer (TBS/ 0.1% Tween 20/ 5% BSA) and then probed with horseradish peroxidase-conjugated secondary antibodies. Proteins were visualized using ECL reagents from BioRad or Millipore (Temecula, CA, USA). Protein expression levels were quantified using Labworks software (UVP, Upland, CA).

**Pancreatic tissue processing and immunostaining**

Pancreases were fixed in 4% PFA overnight at 4°C, washed with PBS, soaked in 30% sucrose, embedded in OCT and sectioned 4µm thick. Sections were washed with TBS/ 0.1% Tween 20, permeabilized in soaking buffer (TBS/ 0.4% Triton X-100) for 30min on ice, and washed with TBS. After blocking with TBS/ 0.2% Triton X-100/ 3% BSA, immunofluorescence staining was performed with primary antibodies diluted in TBS/ 0.2% Tween 20/ 3% BSA at 4°C overnight, followed after washing by secondary antibodies for 1h at room temperature. Sections were stained

for Glp1R or Glut2 and counterstained for insulin and glucagon. Slides were mounted with Vectashield with DAPI (H-1200 Vector Laboratories, Burlingame, CA, USA). Sections were viewed and imaged using Leica DM6000 microscope (Leica Microsystems, Buffalo Grove, IL, USA) with ×20 objective, equipped with OpenLab 5.5 software (Improvision, Coventry, UK).

**Antibodies**

For Western Blotting primary antibodies were used: rabbit anti-MafA (Bethyl A300-611A, RRID:AB_2297116, Montgomery, TX, USA, 1:1000), rabbit anti-Nkx6.1 (Cell Signaling Technology 54551, RRID:AB_2722625, Danvers, MA, USA, 1:1000), rabbit anti-Pdx1 (Cell Signaling Technology 5679, RRID:AB_10706174, 1:1000) and rabbit anti-PARP (loading control) (Cell Signaling Technology 9542, RRID:AB_2160739, 1:1000). For immunofluorescence staining we used mouse anti-Glp1r (Developmental Studies Hybridoma Bank 7F38, RRID:AB_2618101, Iowa, IA, USA, 1:100), rabbit anti-Glut2 (Sigma-Aldrich 07-1402-I, Temecula, CA, USA, 1:300); guinea pig anti-insulin (Abcam ab195956, RRID:AB_2877638, Cambridge, MA, USA, 1:400), mouse anti-glucagon (Sigma-Aldrich G2654, Clone K79bB10, RRID:AB_259852, St. Louis, MO, USA, 1:1000) or rabbit anti-glucagon (Immunostar 20076, AB_572241, Hudson, WI, USA, 1:1000). Secondary donkey antibodies were F(ab)2 fragments conjugated to FITC, Cy3 or Alexa 647 from The Jackson Laboratories (West Grove, PA, USA), and used at dilution of 1:200.

**RNA-seq of mouse islets**

RNA samples from three mice per group were used for RNA-seq (ESM Table 1). Total RNA was extracted from islet samples (176±11 islets per mouse) were using the RNeasy Mini Kit (Qiagen). RNA integrity was confirmed using the Agilent Bioanalyzer 2100 (RIN score range:

6.8–8.9). RNA-seq libraries were prepared using the TruSeq with Ribo-Zero treatment (Illumina, San Diego, CA, USA) to deplete rRNA. cDNA libraries were generated using the NuGEN Ovation kit (NuGEN, Redwood City, CA, USA). Illumina's NextSeq 500 platform was used to generate 75 bp, paired-end reads (64±1.4M reads per sample). Short reads were aligned to the mouse reference genome build GRCm38 (mm10) using the STAR aligner [5]. Between 65 and 75% (average 70%) of the reads mapped uniquely to the mouse genome. The HT-Seq package [6] was used to count the number of fragments aligned to known exonic regions. Gene expression was measured as total fragment counts per gene. Sample clustering of islet RNA-seq using multidimensional scaling largely reflected genotype (ESM Fig. 2). RNA-seq data has been deposited within the Gene Expression Omnibus (GEO) repository, accession number GSE148809.

**RNA-seq of human islets**

RNA-seq data from human pancreatic islets were downloaded from GEO (GSE50244) [7]. Data from 77 samples with available $HbA_{1C}$ values were analyzed. Read counts were normalized via the trimmed mean method prior to differential expression analysis using the edgeR package [8]. One type 2 diabetes sample was excluded as an outlier (GSM1216834); therefore 76 samples were included in this manuscript: 51 from normoglycaemic donors (HbA1c 5.4±0.1; BMI 26±0.3; Age 56±2; 18F/33M), 15 prediabetic (HbA1c 6.1±0.03; BMI 26±1; Age 61±2; 6F/9M), and 10 type 2 diabetic donors (HbA1c 7.5±0.3; BMI 30±1; Age 61±3; 6F/4M). No additional information about the donors or islet morphology was available.

**RNA-seq data analysis**

***Differential expression analysis.*** We performed differential expression analysis using the edgeR package [8]. Differentially expressed genes (DEGs) between two groups were identified at false discovery rate (FDR) < 0.05.

***Rank-rank hypergeometric overlap (RRHO) analysis.*** RRHO is a threshold-free algorithm for comparison of overall strength and patterns of overlap between two independent expression profiles [9, 10]. To compare differential expression gene sets using RRHO, only genes measured in both sets can be included in the analysis, and the rest discarded before transforming the expression profiles into continuous ranked gene lists. Genes are assigned ranks by multiplying the sign of the log2-transformed fold-change by degree of differential expression (the -log10(p-value) from the t-test) so that the most significantly up- and down-regulated genes are given the highest and lowest ranks.

Instead of only determining the degree of overlap for genes exceeding some static significance cutoff, RRHO uses a sliding rank threshold to iteratively (step size = 110) evaluate the significance of overlap (based on the hypergeometric distribution) for the number of genes above the rank threshold in each list. The resulting heatmap is a color-coded matrix of –log10-transformed hypergeometric p-values with both dimensions equal to the length of the ranked lists. Genes up-regulated in both sets (co-upregulated) are in the bottom left quadrant, while genes that are down-regulated in both sets (co-downregulated) are in the top right quadrant. Each pixel represents the significance of overlap for the pair of rank thresholds indicated by its coordinate location, and the pixel with the maximum absolute value indicates the pair of rank thresholds containing the set of differentially expressed genes with the most statistically significant overlap.

***Functional enrichment analysis.*** DEG lists from differential expression analyses and genes in co-expression modules related to traits of interest were functionally annotated using Enrichr [11]. We evaluated each list to identify overrepresentation of Gene Ontology (GO) terms and Kyoto Encyclopedia of Genes and Genomes (KEGG) pathways [12] at FDR < 0.05.

***Mendelian and rare variant enrichment.*** We tested enrichment for genes implicated in Mendelian forms of disease using the GeneOverlap tool for R, with FDR-correction for multiple testing. Three curated lists of genes with pathogenic mutations reported to co-segregate with diabetes or a syndrome associated with increased prevalence of diabetes or primary metabolic phenotype were adapted for analysis [13].

***Common variant enrichment.*** Summary statistics were downloaded from GWAS meta-analysis of type 2 diabetes [14]. We performed gene-level analysis using multi-marker analysis of genomic annotation (MAGMA v1.07), a framework that accounts for linkage disequilibrium (LD) between SNPs [15]. LD was calculated using the 1000 Genomes European reference dataset. First, SNPs were mapped to genes (hg19 genome build, with the MHC region excluded) based on the SNP's presence in the region spanning 5kb upstream and downstream of a gene's start and stop sites. Gene-level analysis was then performed to create aggregate statistics for each gene. GWAS candidates were defined as the set of 198 genes reaching genome-wide significance with a one-to-one mouse ortholog. An expanded set of GWAS genes was also tested, using the full set of 634 genes mapping to 81 established type 2 diabetes risk loci with a one-to-one mouse ortholog. Additional type 2 diabetes associated genes were identified using the NHGRI-EBI GWAS Catalog [16].

***Weighted gene co-expression network analysis (WGCNA)***. We identified sets of highly co-expressed genes using the R package WGCNA [17, 18]. After estimating gene-gene correlations using the biweight midcorrelation, a signed, weighted correlation network was used to identify co-

expression modules composed of highly positively correlated genes. The module eigengene (ME), defined as the first principle component of a module, was used to summarize the expression pattern of a given module. Gene-module connectivity, or module membership, was determined by the correlation between expression of each gene with that of each ME.

WGCNA module enrichment was performed for type 2 diabetes GWAS using the Marker Set Enrichment Analysis (MSEA), from the Mergeomics R package [19]. MSEA examines whether the modules are enriched for disease causal genes informed by human type 2 diabetes GWAS using a Chi-square like statistic. Here, we used islet eQTLs to map SNPs to genes. We filtered for potential linkage disequilibrium r2>0.5 to remove redundancies. MSEA can then utilize the disease association p-values for the mapped SNPs from the type 2 diabetes GWAS from DIAGRAM [20]. The disease association p-values of the SNPs representing the modules were then compared with SNPs mapped to random genes to see whether the modules have SNPs that show stronger type 2 diabetes associations than random genes.

***Cell type enrichment analysis.*** Lists of canonical and computationally-inferred genes with high specificity (mouse specificity < 0.05) for each of the nine pancreatic cell types (alpha, beta, delta, gamma, epsilon, pancreatic progenitor, ductal, acinar and pancreatic stellate) and four non-pancreatic cell types (peri-islet Schwann, macrophages, pericytes and endothelial cells) were downloaded from the PanglaoDB cell marker compendium [21]. We tested enrichment of each set of cell-type markers among the up- or down-regulated DEGs or co-expression modules using Fisher's exact test as implemented in the GeneOverlap R tool [22] with FDR-correction for multiple testing. For DEG lists, we separately examined overrepresentation in up- and down-regulated genes. For co-expression modules, we restricted analysis to genes with module membership > 0.5 and FDR p-value < 0.05. Results are presented as a heatmap, with color

showing -log10 FDR p-value and the odds ratio reported for $q < 0.05$.

***Deconvolution of bulk islet RNA-seq***. We downloaded single cell RNA-Seq data for mouse islets from GEO (GSM2230762) as our reference dataset, and utilized CibersortX [23] as our tool for deconvolution for each genotype bulk islet RNA-Seq results. We utilized the Impute Cell Fractions function and ran with the recommended 100 permutations. Each mouse genotype had an n=3, to showcase results which were representative of each group, we used the average gene expression value for each gene across the n of 3, prior to running deconvolution.

***Transcription factor (TF) network analysis and gene regulatory network analysis.***

For the TF network analysis, we utilized the Enrichr tool with the TF-Gene Cooccurrence extension, where the overlapping DEGs between hIAPP and type 2 diabetes were utilized as input. To identity additional non-TF regulators, we utilized the key driver analysis (KDA) function from the Mergeomics R package [19] in order to identify the key regulatory genes for both DEG sets and for the 15 co-expression modules using a Bayesian gene network. The Bayesian network contains both islet and brain gene-gene relationships due to similarity in the gene regulatory relationships as well as the importance of protein misfolding in both tissues. A key driver is defined as a gene that is connected to a large number of genes from a DEG list or co-expression module, in comparison to the expected number for a randomly selected gene within a Bayesian network based on a Chi-square like statistic $\chi = \frac{O-E}{\sqrt{E}-\kappa}$, where O and E represent the observed and expected ratios of genes from disease-associated gene sets in a hub subnetwork, and $E = \frac{N_k N_p}{N}$ is estimated using the hub degree $N_k$, disease gene set size $N_p$ and the order of the full network $N$. Statistical significance of the disease-gene enriched KDs is estimated by permuting the network gene labels 10000 times and estimating the P-value based on the null distribution.

# ESM References


[1] Janson J, Soeller WC, Roche PC, et al. (1996) Spontaneous diabetes mellitus in transgenic mice expressing human islet amyloid polypeptide. Proc Natl Acad Sci U S A 93(14): 7283-7288. 10.1073/pnas.93.14.7283

[2] Gurlo T, Costes S, Hoang JD, Rivera JF, Butler AE, Butler PC (2016) beta Cell-specific increased expression of calpastatin prevents diabetes induced by islet amyloid polypeptide toxicity. JCI Insight 1(18): e89590. 10.1172/jci.insight.89590

[3] Huang CJ, Haataja L, Gurlo T, et al. (2007) Induction of endoplasmic reticulum stress-induced beta-cell apoptosis and accumulation of polyubiquitinated proteins by human islet amyloid polypeptide. Am J Physiol Endocrinol Metab 293(6): E1656-1662. 10.1152/ajpendo.00318.2007

[4] Rivera JF, Costes S, Gurlo T, Glabe CG, Butler PC (2014) Autophagy defends pancreatic beta cells from human islet amyloid polypeptide-induced toxicity. J Clin Invest 124(8): 3489-3500. 10.1172/JCI71981

[5] Dobin A, Davis CA, Schlesinger F, et al. (2013) STAR: ultrafast universal RNA-seq aligner. Bioinformatics 29(1): 15-21. 10.1093/bioinformatics/bts635

[6] Anders S, Pyl PT, Huber W (2015) HTSeq--a Python framework to work with high-throughput sequencing data. Bioinformatics 31(2): 166-169. 10.1093/bioinformatics/btu638

[7] Fadista J, Vikman P, Laakso EO, et al. (2014) Global genomic and transcriptomic analysis of human pancreatic islets reveals novel genes influencing glucose metabolism. Proc Natl Acad Sci U S A 111(38): 13924-13929. 10.1073/pnas.1402665111

[8] Law CW, Chen Y, Shi W, Smyth GK (2014) voom: Precision weights unlock linear model analysis tools for RNA-seq read counts. Genome Biol 15(2): R29. 10.1186/gb-2014-15-2-r29

[9] Cahill KM, Huo Z, Tseng GC, Logan RW, Seney ML (2018) Improved identification of concordant and discordant gene expression signatures using an updated rank-rank hypergeometric overlap approach. Sci Rep 8(1): 9588. 10.1038/s41598-018-27903-2

[10] Plaisier SB, Taschereau R, Wong JA, Graeber TG (2010) Rank-rank hypergeometric overlap: identification of statistically significant overlap between gene-expression signatures. Nucleic Acids Res 38(17): e169. 10.1093/nar/gkq636

[11] Chen EY, Tan CM, Kou Y, et al. (2013) Enrichr: interactive and collaborative HTML5 gene list enrichment analysis tool. BMC Bioinformatics 14: 128. 10.1186/1471-2105-14-128

[12] Ogata H, Goto S, Fujibuchi W, Kanehisa M (1998) Computation with the KEGG pathway database. Biosystems 47(1-2): 119-128. 10.1016/s0303-2647(98)00017-3

[13] Fuchsberger C, Flannick J, Teslovich TM, et al. (2016) The genetic architecture of type 2 diabetes. Nature 536(7614): 41-47. 10.1038/nature18642

[14] Mahajan A, Taliun D, Thurner M, et al. (2018) Fine-mapping type 2 diabetes loci to single-variant resolution using high-density imputation and islet-specific epigenome maps. Nat Genet 50(11): 1505-1513. 10.1038/s41588-018-0241-6

[15] de Leeuw CA, Mooij JM, Heskes T, Posthuma D (2015) MAGMA: generalized gene-set analysis of GWAS data. PLoS Comput Biol 11(4): e1004219. 10.1371/journal.pcbi.1004219

[16] Buniello A, MacArthur JAL, Cerezo M, et al. (2019) The NHGRI-EBI GWAS Catalog of published genome-wide association studies, targeted arrays and summary statistics 2019. Nucleic Acids Res 47(D1): D1005-D1012. 10.1093/nar/gky1120

[17] Langfelder P, Horvath S (2007) Eigengene networks for studying the relationships between co-expression modules. BMC Syst Biol 1: 54. 10.1186/1752-0509-1-54

[18] Langfelder P, Horvath S (2008) WGCNA: an R package for weighted correlation network analysis. BMC Bioinformatics 9: 559. 10.1186/1471-2105-9-559

[19] Shu L, Zhao Y, Kurt Z, et al. (2016) Mergeomics: multidimensional data integration to identify pathogenic perturbations to biological systems. BMC Genomics 17(1): 874. 10.1186/s12864-016-3198-9

[20] Morris AP, Voight BF, Teslovich TM, et al. (2012) Large-scale association analysis provides insights into the genetic architecture and pathophysiology of type 2 diabetes. Nature genetics 44(9): 981
[21] Franzen O, Gan LM, Bjorkegren JLM (2019) PanglaoDB: a web server for exploration of mouse and human single-cell RNA sequencing data. Database (Oxford) 2019. 10.1093/database/baz046
[22] Shen L (2014) GeneOverlap: An R package to test and visualize gene overlaps. R Package
[23] Newman AM, Steen CB, Liu CL, et al. (2019) Determining cell type abundance and expression from bulk tissues with digital cytometry. Nature Biotechnology 37(7): 773-782. 10.1038/s41587-019-0114-2

**ESM Table 1** Characteristics of mice and samples subjected to sequencing

| Mice genotype | Age, weeks | Body weight, g | Blood glucose, mmol/l | Number of islets | RIN | Number of reads | Uniquely mapped reads, % |
|---|---|---|---|---|---|---|---|
| WT | 10 | 25 | 4.1 | 240 | 8.6 | 64418000 | 74 |
| WT | 9 | 25 | 5.7 | 195 | 8.7 | 77329568 | 70 |
| WT | 9 | 24 | 5.0 | 169 | 7.2 | 72279923 | 70 |
| rIAPP | 9 | 23 | 5.5 | 117 | 8.6 | 59223155 | 70 |
| rIAPP | 9 | 24 | 3.3 | 136 | 8.4 | 68674516 | 68 |
| rIAPP | 9 | 24 | 3.3 | 193 | 8.3 | 61936671 | 69 |
| hIAPP | 9.5 | 23 | 5.1 | 177 | 8.6 | 62276209 | 68 |
| hIAPP | 9.5 | 21 | 4.5 | 118 | 8.5 | 66919602 | 67 |
| hIAPP | 9 | 23 | 5.7 | 137 | 6.8 | 57542237 | 71 |
| hCAST | 10 | 24 | 3.4 | 240 | 8.8 | 59141131 | 71 |
| hCAST | 9 | 25 | 4.1 | 230 | 8.7 | 61697556 | 72 |
| hCAST | 9 | 24 | 3.3 | 212 | 8.9 | 61396560 | 71 |
| hIAPP:hCAST | 9.5 | 21 | 5.0 | 162 | 8.4 | 60746686 | 70 |
| hIAPP:hCAST | 9.5 | 24 | 4.4 | 147 | 8.3 | 61905765 | 71 |
| hIAPP:hCAST | 9 | 22 | 4.8 | 173 | 6.8 | 64789817 | 74 |

Blood glucose and body weight were measured after overnight fast right before islet isolation

**ESM Table 2** Characteristics of mice used to generate data for Fig. 4 a, b

| Genotype | Age, weeks | Body weight, g | Blood glucose, mmol/l | Western blot and PCR | IF staining |
|---|---|---|---|---|---|
| WT | 9 | 23 | 3.1 | x | |
| WT | 9 | 25 | 3.2 | x | |
| WT | 9 | 25 | 3.2 | x | |
| WT | 11 | 24 | 4.1 | | x |
| WT | 11 | 23 | 3.3 | | x |
| rIAPP | 9 | 27 | 3.6 | x | |
| rIAPP | 9 | 30 | 3.4 | x | |
| rIAPP | 9 | 28 | 3.7 | x | |
| rIAPP | 9.5 | 24 | 3.1 | | x |
| rIAPP | 9.5 | 24 | 2.9 | | x |
| hIAPP | 9 | 25 | 3.2 | x | |
| hIAPP | 9 | 27 | 6.6 | x | |
| hIAPP | 9 | 22 | 2.7 | x | |
| hIAPP | 9.5 | 22 | 4.0 | | x |
| hIAPP | 9.5 | 22 | 4.4 | | x |

Blood glucose and body weight were measured after overnight fast right before tissue collection

**ESM Table 3** Characteristics of mice used in IPGTT

| Group | n | Body weight, g | Blood glucose, mmol/l |
|---|---|---|---|
| WT | 14 | 25.6 ± 0.3 | 3.7 ± 0.2 |
| rIAPP | 8 | 26.3 ± 0.3 | 2.9 ± 0.2 |
| hIAPP | 5 | 26.5 ± 0.5 | 3.8 ± 0.2 |

Data is presented as mean ± SEM

Blood glucose and body weight were measured after overnight fast right before dextrose injection

**ESM Table 4** Characteristics of mice used for beta cell mass assessment

| Group | n | Body weight, g | Blood glucose, mmol/l | Beta cell mass, mg |
|---|---|---|---|---|
| WT | 4 | 24.0 ± 0.4 | 4.1 ± 0.1 | 3.1 ± 0.8 |
| rIAPP | 4 | 24.0 ± 0.2 | 3.8 ± 0.4 | 2.9 ± 0.4 |
| hIAPP | 4 | 23.0 ± 0.3 | 4.3 ± 0.3 | 2.7 ± 0.4 |
| hCAST | 4 | 24.0 ± 0.2 | 4.1 ± 0.2 | 2.4 ± 0.7 |
| hCAST:hIAPP | 4 | 23.0 ± 0.5 | 4.2 ± 0.1 | 2.4 ± 0.3 |

Data is presented as mean ± SEM

Blood glucose and body weight were measured after overnight fast right before tissue collection

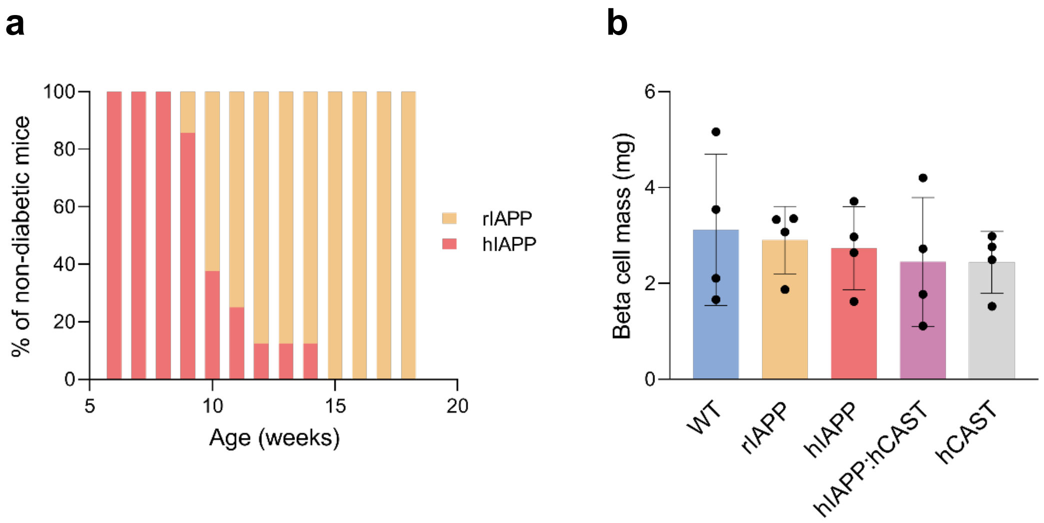


**ESM Fig. 1.** Diabetes development and beta cell mass. (**a**) Diabetes was monitored by weekly measurement of tail vein blood glucose after overnight fast in 6-18 weeks-old mice. A mouse was considered diabetic if fasting blood glucose was >6.9mmol/l. Data is % of non-diabetic mice; n=5-16 for rIAPP and 5-14 for hIAPP group per column. (**b**) Beta cell mass in 9-weeks-old mice with fasting glucose and body weight matching to mice used for RNA-seq analysis was comparable in all groups. Mice characteristics are presented in ESM Table 4.

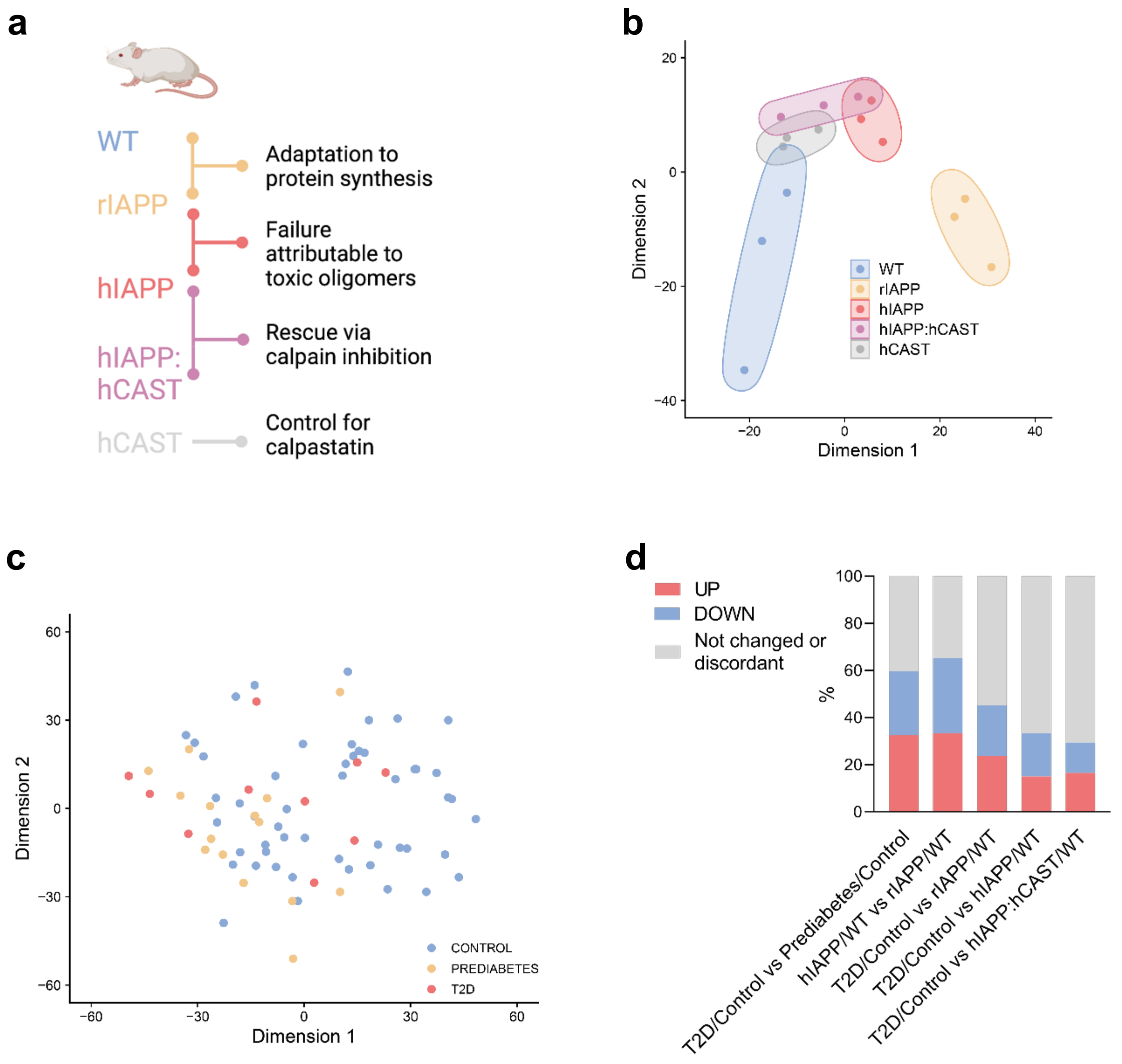


**ESM Fig. 2**. (**a**) Schematic depicting experimental and control groups, with rationale and expected output for each comparison. Created with Biorender.com. (**b**) Multidimensional scaling map of islet profiles shows clustering of samples is largely influenced by genotype of mice. (**c**) Multidimensional scaling map of human islet samples. (**d**) The proportion of genes concordantly up- and down-regulated obtained from RRHO analysis of listed pairs of comparisons.

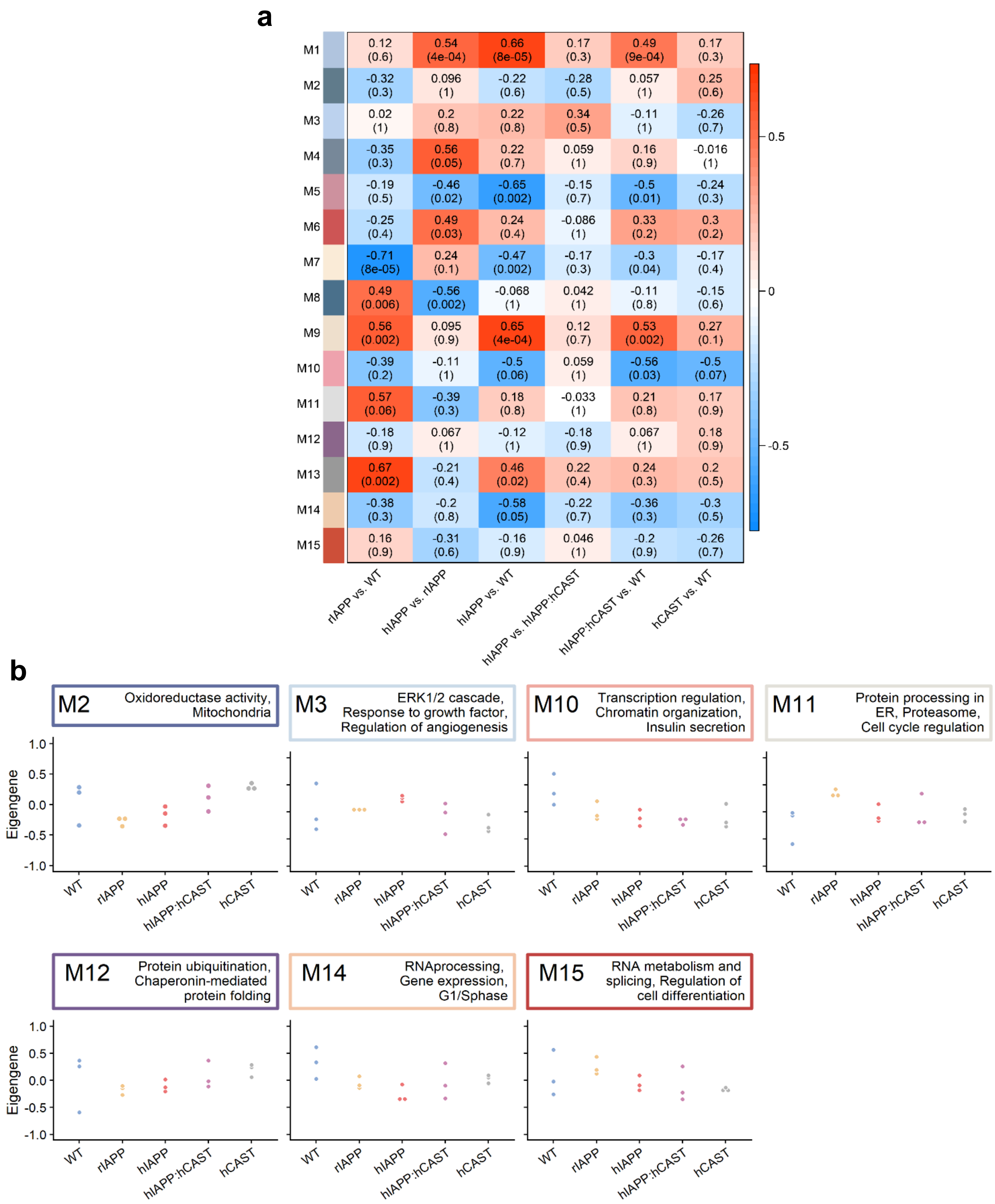


**ESM Fig. 3.** Co-expression network construction and analysis. (**a**) Heatmap displays correlation coefficient between module expression and pairwise islet comparisons. Significant module level perturbations are denoted (*, $q < 0.05$). (**b**) Plots of ME trajectory by sample group, for seven modules not shown in the main text.

a

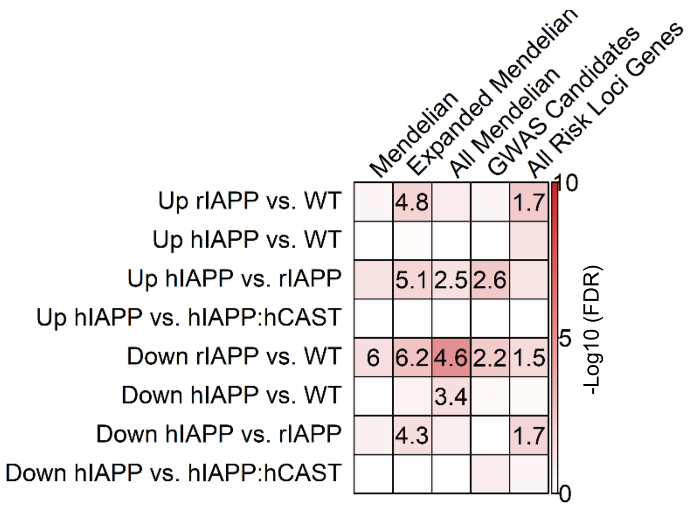


b

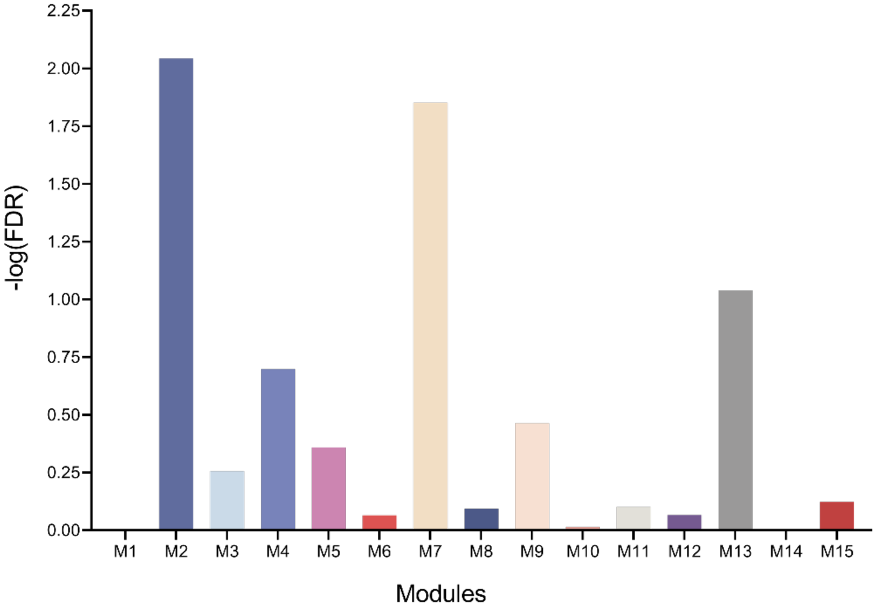


**ESM Fig. 4.** (**a**) Common and rare variant enrichment of DEGs. (**b**) Type 2 diabetes GWAS enrichment for 15 WGCNA co-expression modules.

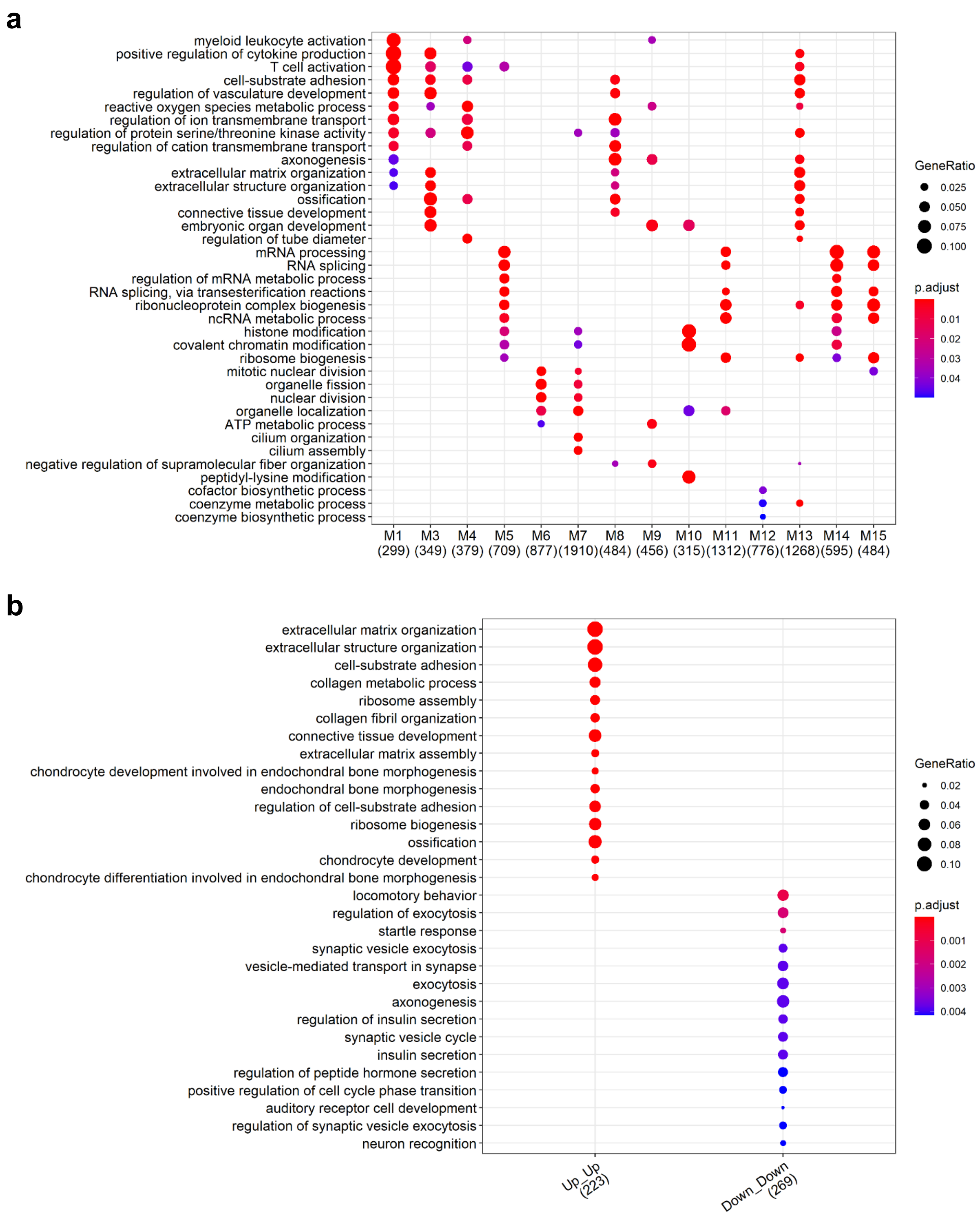


**ESM Fig. 5.** (**a**) GO biological process term enrichment of co-expression modules. Enrichment analysis and visualization were performed using ClusterProfiler. No overrepresented terms were identified for M2 (excluded from visualization). (b) Functional annotation of differentially expressed genes (FDR < 0.05) that are upregulated (Up_Up) or downregulated (Down_Down) in both hIAPP/WT and rIAPP/WT. GO biological process term enrichment of DEG sets.

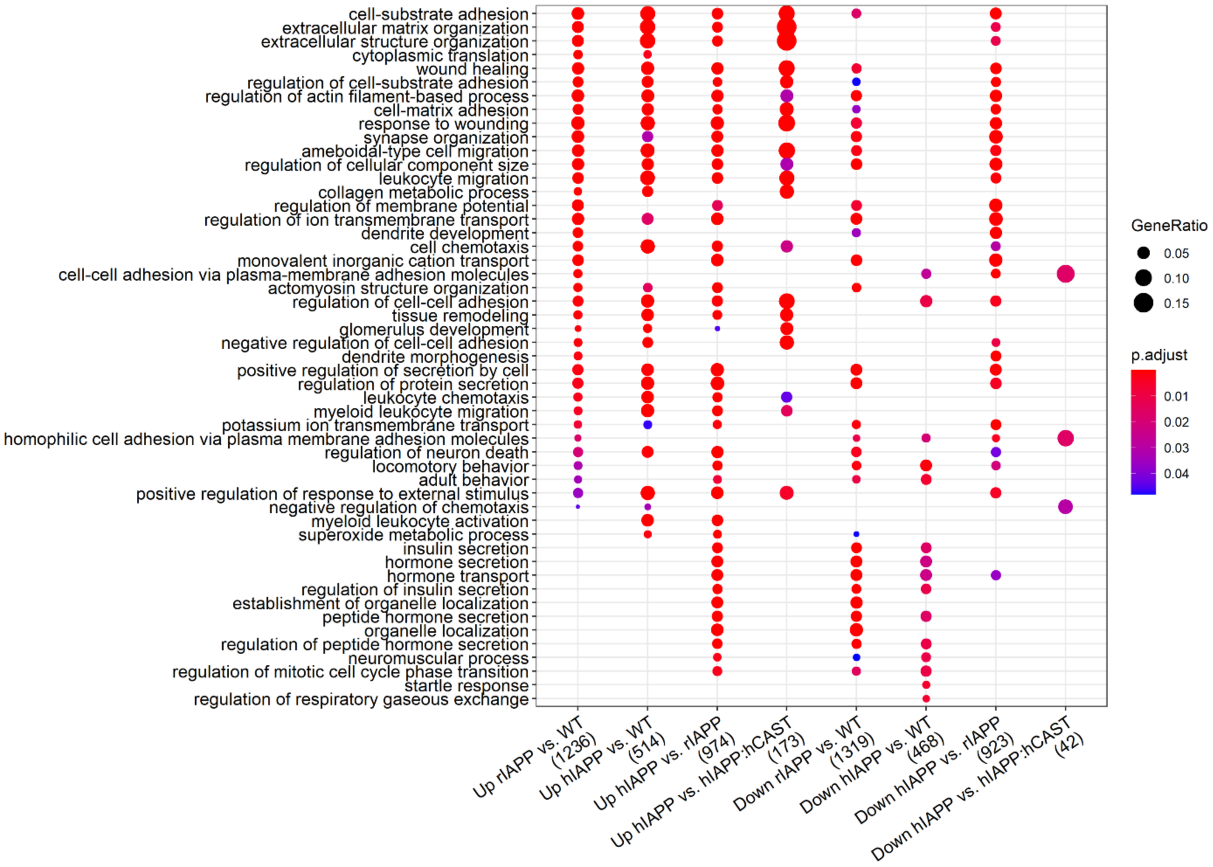


**ESM Fig. 6.** Functional annotation of differentially expressed genes (FDR < 0.05). GO biological process term enrichment of DEG sets. Enrichment analysis and visualization were performed using ClusterProfiler. No enrichment analysis was performed for DEGs identified between hCAST and WT islets, as there was only one gene identified (*Tram1l1*).

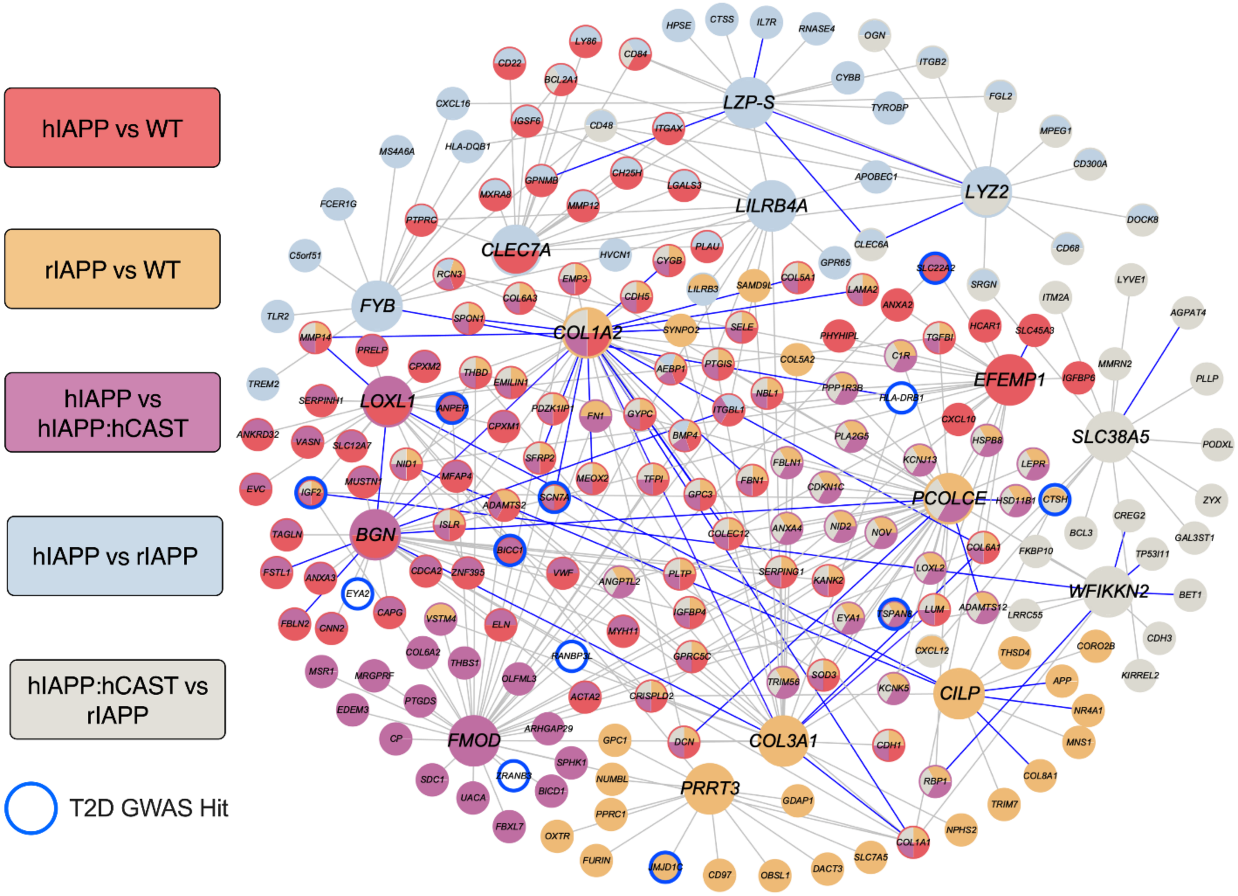


**ESM Fig. 7.** Gene-gene regulatory subnetwork (Bayesian Network) and top key drivers of differentially expressed genes from IAPP sequencing between the various genotypes (color indicates comparison(s) in which a gene is differentially expressed; FDR < 0.05). Large nodes indicate key driver genes.
